# The Ras/ERK signaling pathway couples antimicrobial peptides to mediate resistance to dengue virus in *Aedes* mosquitoes

**DOI:** 10.1371/journal.pntd.0008660

**Published:** 2020-08-31

**Authors:** Wen-Quan Liu, Si-Qi Chen, Hao-Qiang Bai, Qi-Mei Wei, Sheng-Nan Zhang, Chen Chen, Yi-Han Zhu, Tang-Wei Yi, Xiao-Pu Guo, Si-Yuan Chen, Meng-Jie Yin, Chen-Feng Sun, Shao-Hui Liang

**Affiliations:** 1 Department of Parasitology, School of Basic Medical Sciences, Wenzhou Medical University, Wenzhou, China; 2 School of the 1^st^ Clinical Medical Sciences, Wenzhou Medical University, Wenzhou, China; Institut Pasteur, FRANCE

## Abstract

*Aedes* mosquitoes can transmit dengue and several other severe vector-borne viral diseases, thereby influencing millions of people worldwide. Insects primarily control and clear the viral infections via their innate immune systems. Mitogen-Activated Protein Kinases (MAPKs) and antimicrobial peptides (AMPs) are both evolutionarily conserved components of the innate immune systems. In this study, we investigated the role of MAPKs in *Aedes* mosquitoes following DENV infection by using genetic and pharmacological approaches. We demonstrated that knockdown of ERK, but not of JNK or p38, significantly enhances the viral replication in *Aedes* mosquito cells. The Ras/ERK signaling is activated in both the cells and midguts of *Aedes* mosquitoes following DENV infection, and thus plays a role in restricting the viral infection, as both genetic and pharmacological activation of the Ras/ERK pathway significantly decreases the viral titers. In contrast, inhibition of the Ras/ERK pathway enhances DENV infection. In addition, we identified a signaling crosstalk between the Ras/ERK pathway and DENV-induced AMPs in which defensin C participates in restricting DENV infection in *Aedes* mosquitoes. Our results reveal that the Ras/ERK signaling pathway couples AMPs to mediate the resistance of *Aedes* mosquitoes to DENV infection, which provides a new insight into understanding the crosstalk between MAPKs and AMPs in the innate immunity of mosquito vectors during the viral infection.

## Author summary

*Aedes* mosquitoes are the vectors that can transmit dengue fever and several other severe vector-borne viral diseases in the human population. Insects control and clear viral infections via their innate immune systems. Studies of the genes and molecules in mosquitos that control pathogen infection may provide insights into finding novel targets for controlling the transmission of mosquito-borne diseases. In this study, we used genetic and pharmacological approaches to investigate the role of MAPKs in *Aedes* mosquitoes following DENV2 infection in vitro and in vivo. Activation of the Ras/ERK pathway via insulin treatment and overexpression of Ras significantly decreased the DENV titers, whereas inhibition of the Ras/ERK pathway by either U0126 treatment or by Ras knockdown enhanced DENV infection. We demonstrated that the Ras/ERK signaling couples AMPs to mediate the resistance of *Aedes* mosquitoes to DENV infection, which reveals a novel antiviral mechanism regulated by multiple innate immune components in mosquitoes.

## Introduction

Dengue is one of the most common and rapidly spreading mosquito-borne viral diseases, with approximately 50 million to 100 million cases being reported annually that have been found in more than 100 countries located in the tropical and subtropical regions of the world [1, 2]. *Aedes* mosquitoes, including *Aedes aegypti* (*Ae*. *aegypti*) and *Aedes albopictus* (*Ae*. *albopictus*), are the principal vectors for the transmission of dengue virus (DENV) [3, 4]. Moreover, *Aedes* mosquitoes transmit other important arboviruses, such as chikungunya virus (CHIKV), yellow fever virus (YFV) and Zika virus (ZIKV) [5, 6]. Due to a lack of effective drugs and the limited application of licensed vaccines, vector control has remained the most important approach to preventing epidemics of dengue and other mosquito-borne diseases [4, 7]. However, the traditional strategies used to control mosquitoes are facing great challenges due to an expansion of their geographic distribution and an increase in the prevalence of insecticide resistance [6–8]. Importantly, studies of the mosquito-associated mechanisms that mediate the resistance to pathogen infections can aid in the development of novel strategies to control mosquito-borne diseases [9–12].

Insects, such as *Drosophila* and mosquitoes, primarily rely on their innate immune systems to limit and clear pathogens following an infection [13–15]. The innate immune response is triggered by the recognition of pathogen-associated molecular patterns (PAMPs) through host pattern recognition receptors (PRRs), resulting in the activation of immune signaling pathways and the production of effector molecules to suppress pathogen replication [13, 16]. The immune signaling pathways, including Toll, immune deficiency (IMD), JAK-STAT and RNAi pathways, are highly conserved between *Drosophila* and mosquitoes [17–19]. In *Drosophila*, both the Toll and IMD pathways are primarily activated by bacteria and fungi to regulate the expression of antimicrobial peptides (AMPs) [14]. In *Ae*. *aegypti* mosquitoes, the Toll pathway and complement-like system are activated by DENV infection and thus induce the production of AMPs to limit virus propagation [18, 20]. In addition, the JAK-STAT and RNAi pathways are considered to play an important role in antiviral immunity in both *Drosophila* and mosquitoes [17, 21, 22].

Mitogen-activated protein kinases (MAPKs) are among the most conserved signaling cascades and regulate the host cellular responses to a variety of extracellular stimuli, such as growth factors, cytokines, oxidative damage and pathogens infection, in many model systems [23–25]. In mammalian cells, 14 MAPKs have been characterized. Three conventional MAPKs, including extracellular signal-regulated kinase (ERK), c-Jun N-terminal kinase (JNK) and p38, have been extensively studied with respect to the innate immunity [24, 26]. The orthologs of mammalian MAPKs having been identified in *Caenorhabditis elegans*, *Drosophila* and *Anopheles* mosquitoes [23, 24, 26]. The MAPK signaling pathway regulates a variety of cellular processes, including growth, metabolism, apoptosis and innate immune responses in insects [23, 27, 28]. For example, the *Drosophila* ERK/MAPK pathway has been shown to be involved in maintaining midgut homeostasis and regeneration [27, 29, 30], and in mediating innate immunity against viral infection [31, 32], while JNK and p38 participate in the insect host defense against bacterial and fungal infections [33, 34].

To date, 17 MAPK orthologs, including ERK, JNK, and p38, have been identified in *Anopheles gambiae* (*An*. *gambiae*) mosquitoes and shown to regulate their metabolism, longevity and response to *Plasmodium* parasite infection [26, 35, 36]. Inhibition of JNK in the *Ae*. *albopictus* mosquito cell line C6/36 blocks the internalization of fluorescein-labeled bacteria [37]. In addition, the MAPK pathway was identified as a core signaling pathway activated by DENV infection in *Ae*. *aegypti* mosquitoes [38]. However, the role of MAPKs in the interaction of *Aedes* mosquito hosts and DENV remains unclear. In this study, we investigated the function of MAPKs in both the cells and midguts of *Aedes* mosquitoes following DENV2 infection. We identified that ERK, but not JNK or p38, is involved in controlling DENV2 replication in *Aedes* mosquito cells. The Ras/ERK pathway play a role in resisting DENV2 infection in *Aedes* mosquitoes. Furthermore, we demonstrated that the Ras/ERK pathway influences the AMPs expression and couples defensin C to mediate the resistance of *Aedes* mosquitoes against DENV.

## Methods

### Ethics statement

*Ae*. *albopictus* mosquito (Foshan strain) was the only experimental animal used in our study, which did not have animal ethical issues. Human blood was donated by the healthy volunteers who provided written informed consent. The use of human blood products in this study was approved by the ethics committee of Wenzhou Medical University (Permit Number: 2019–108).

### Antibodies and reagents

Monoclonal anti-phosphorylated MAPKs (ERK1/2, JNK, and p38) antibodies and polyclonal anti-MAPKs antibodies were obtained from Cell Signaling Technology (Danvers, MA). Mouse anti-DENV2 envelope monoclonal antibody, Goat anti-mouse IgG conjugated with Alexa-488 fluorochrome, anti-β-actin antibody, and horseradish peroxidase-conjugated polyclonal rabbit anti-mouse IgG were purchased from GeneTex (Irvine, CA). Recombinant human insulin and the MEK1/2 inhibitor U0126 were obtained from MedChemExpress (Monmouth Junction, NJ). The BCA Protein Assay Kit and SuperSignal West Pico Chemiluminescent Detection kit were purchased from Thermo Fisher Scientific (Waltham, MA). All other chemicals and reagents were obtained from Sigma-Aldrich.

### Bioinformatics analysis

The gene sequences encoding the MAPK signaling factors ERK, JNK and p38 of mosquitoes were obtained from NCBI and VectorBase (https://www.vectorbase.org/) by using KEGG and BLAST analyses. The sequences of the MAPK signaling factors of *Dm*. *melanogaster* were obtained from Flybase (http://flybase.org). The MAPK sequences were aligned using Clustal X, and an unrooted phylogenetic tree was built with MEGA 7 software by using the neighbor-joining method. The bootstrap consensus tree was inferred from 1000 replicates. The sequence accession numbers of the MAPK signaling factors from *Ae*. *albopictus*, *Ae*. *aegypti*, *An*. *gambiae*, *Culex quinquefasciatus* (*Cx*. *quinquefasciatus*) and *Dm*. *melanogaster* are indicated in the phylogenetic analysis. The siRNAs for *MAPKs* and *AMPs* genes were designed and synthesized by Genepharma (Shanghai), and *GFP* siRNA was used as a control (S1 Table).

### Cell culture, treatment and viral infection

C6/36 and Aag2 cells were cultured at 28°C in RPMI-1640 medium (Gibco) and Schneider’s medium (Invitrogen), respectively. All media were supplemented with 10% heat-inactivated fetal bovine serum. The DENV2 New Guinea C strain was propagated in C6/36 cells as described previously [39]. For the signaling assay, 2×10^5^ C6/36 or Aag2 cells were transferred to 24-well plates and cultured overnight. The cells were stimulated with serum-free media containing several concentrations of human insulin, U0126, or insulin buffer containing U0126, and PBS-treated cells were used as controls. After incubating for 30 min, the culture media were removed. Then, cells were infected with DENV2 at a multiplicity of infection (MOI) of 0.1 and harvested after 6 to 48 hours post-infection (h.p.i) for subsequent analyses.

### Mosquito rearing, feeding and oral infection

*Ae*. *albopictus* mosquitoes were maintained under a 12-hour light/dark cycle at 27°C and 85% humidity. The adult mosquitoes were maintained in a cage and provided with cotton pads soaked in a 10% sucrose solution. Mosquito feeding with artificial bloodmeals was performed according to the previous studies [40, 41]. The artificial blood meal is composed of 50% human red blood cells (RBCs) and 50% saline (10 mM NaHCO3 and 15 mM NaCl, 1 mM ATP, pH 7.0). Female adult mosquitoes aged 6–8 days were fed with artificial blood meals supplemented with insulin or U0126 by using Parafilm membranes and water-jacketed artificial feeders at 37°C for 2 hours. Then, the blood-fed mosquitoes were transferred into a clean carton and maintained with a 10% sucrose solution.

For oral infection with DENV2, female adult mosquitoes were deprived of sugar overnight and water for 8 h prior to the viral infection. DENV2-infected cell suspension with a titer of 3×10^7^ plaque-forming unit (PFU)/mL was mixed with the artificial blood meals at a 1:1 ratio (final titer of DENV2 is 1.5 ×10^7^ PFU/mL) and incubated in 37°C water bath for 30 min. Insulin and U0126 were separately added into the viral blood mixture in the final concertation of 500 μM and 34 μM before feeding. An equivalent volume of PBS was taken as a control.

### Sample preparation, RNA extraction and quantitative PCR

Female adult mosquitoes from each treatment were cold anesthetized on ice, surfaced sterile in 75% ethanol, and washed twice with sterile H_2_O. Then, midguts of mosquitoes were dissected and extensively washed for at least 5 times to remove the undigested blood. The dissected midguts were transferred into a 1.5 mL sterile tube containing 250 μL PBS and homogenized on ice for 1 min. The tissue homogenate was used for RNA extraction.

Total RNA was isolated from the cultured cells or the midgut homogenate using TRIzol reagent (Invitrogen) and treated with DNase (Invitrogen) according to the manufacturer's protocol. Then, the cDNA was generated from total RNA using HiScript II Q Select RT SuperMix (Vazyme Biotech, Nanjing, China). qPCR was performed with an ABI7300 Fluorescent Quantitative PCR System (Applied Biosystems, CA) using the ChamQ SYBR qPCR Master Mix (Vazyme Biotech, Nanjing, Jiangsu). The *Ae*. *albopictus* ribosomal S7 (*Rps7*) and *Ae*. *aegypti* ribosomal S17 (*Rps17*) genes were used as the endogenous controls. The levels of DENV2 mRNA and *AMP* genes expression in the midguts were determined using an absolute quantification qPCR method [42, 43]. A standard curve between copy number and CT value was established by using a series of diluted (1×10^2^ to 1×10^7^) recombinant plasmid standards containing the target gene as a template. The levels of both DENV mRNA and *AMPs* gene expression were presented in logarithmic scale. Primers for real-time qPCR amplification were designed and are listed in S2 Table.

### Western blotting analysis

Protein extraction and Western blotting analyses were performed as previously described with slight modifications [41]. Briefly, the cells and the dissected midguts from each treatment were collected and washed with ice-cold PBS, then lysed in 100 μL RIPA lysis buffer with a protease inhibitor cocktail (APExBIO Tech., Boston, MA). The lysates were centrifuged at 12,000 g for 10 min, the supernatants were collected, and the protein concentrations were measured using a BCA protein assay kit. Then, equivalent concentrations of proteins were mixed with protein loading buffer and heated to 95°C for 10 min. The protein samples were separated on 10% SDS-PAGE polyacrylamide gels and then subjected to Western blotting. Rabbit anti-phospho-MAPK (ERK, JNK, and p38) monoclonal antibodies (1:2000) and anti-MAPK polyclonal antibodies (1:1000) were used to detect phosphorylated MAPK and total MAPK proteins. After washing 3 times, the membranes were incubated with a 1:2000 dilution of HRP-conjugated goat anti-rabbit IgG at room temperature for 1 hour. Each membrane was incubated with SuperSignal West Pico Chemiluminescent Reagent for 5 min to visualize the antibody-bound proteins. The densitometric analysis of the Phospho-ERK levels and total ERK levels were performed by using the ImageJ software. The levels of phosphorylated and total MAPKs were separately normalized to that of β-actin. The fold-change was indicated as the level of induction in each treatment compared with the control.

### Gene knockdown and overexpression

Gene knockdown was performed using the RNAi method. In brief, *Aedes* mosquito cells (C6/36 and Aag2) were seeded into 12-well plates with serum-free media. Then, the cells were transfected with small interfering RNAs (siRNAs) using Lipofectamine 2000 Transfection Reagent (Invitrogen) according to the manufacturer’s protocol. After siRNA transfection for 48 h, the cell samples were collected for qPCR or Western blotting analysis.

For Ras overexpression, the full-length cDNA of the *Ae*. *albopictus* Ras-encoding gene carrying a FLAG tag was amplified by PCR and then cloned into the vector pIB-V5 (Invitrogen). The recombinant pIB-Ras plasmid was confirmed by sequencing. Cells were seeded into 12-well plates overnight and transfected with pIB-Ras using TransIT DNA Transfection Reagent (Mirus). The cells transfected with the pIB-V5 vector were used as the control. Ras protein expression was detected by mouse anti-FLAG monoclonal antibody (GeneTex). A horseradish peroxidase-conjugated polyclonal rabbit anti-mouse IgG was used as a secondary antibody in Western blotting assays.

### Immunofluorescence assay

C6/36 and Aag2 cells (2×10^6^ and 1×10^6^, respectively) were separately plated on coverslips in 12-well culture plates overnight. After treatment, cells were infected with DENV2 (MOI = 0.1) for 36 h, and fixed with 4% (vol/vol) formaldehyde for 30 min. The slides were washed with PBS buffer containing 0.1% Triton X-100 (PBS-T) for 10 min and blocked with 5% BSA in PBS-T for 1 h. Then, the samples were incubated with a mouse anti-DENV2 E protein monoclonal antibody at a 1:200 dilution in 5% BSA /PBS-T overnight at 4°C. After washing for 3 times, the cells were washed with PBS-T three times for 10 min each and incubated with Alexa-488 fluorochrome anti-mouse secondary antibody (GeneTex) at a 1:1000 dilution for 1 h at room temperature. The cells were then incubated with DAPI for 10 min to stain the nucleus, and approximately 1×10^4^ immunostained cells with DAPI staining on each side were examined under a Nikon C2 Plus confocal microscope. Each imaging filed contained a minimum of 100 cells from three different sites and were analyzed by using the automated image analysis software. The percentage of DENV infection was calculated by determining the ratio of viral E antigen-positive cells in the total number of cells in three independent experiments.

### Plaque assays

Plaque assays were conducted as previously described [20, 44]. In brief, mosquitoes were dissected on serial days post-infection. Midguts from the individual mosquitoes were homogenized in 200μL PBS buffer followed by centrifugation at 7500 g for10 min at 4˚C. The supernatants were collected for the plaque assay. BHK-21 cells were seeded into a 24-well plate and cultured for overnight. The midgut lysates were inoculated onto the cells with a ten-fold dilution (from10^0^ to 10^−4^) in duplicates. After two hours of incubation at 37˚C, cell monolayers were washed for twice with 1 mL PBS per well. An overlay DMEM media consisting of 2% FBS and melting Agarose were added in each well, and the plates were incubated for 5 days at 37°C. Finally, the plates were stained with 1% crystal violet for 20 minutes and the number of plaques was counted. The viral titers were presented as plaque-forming units per midgut (PFU/midgut).

### Statistical analyses

The statistical analyses were performed using GraphPad Prism8.0 software. The data were assayed for normality using the Shapiro-Wilk normality test. In the cell experiments, the data from the experimental group and the control group satisfying the normal distribution were compared by the Student's t-test. In the mosquito infection experiments, the distribution-free quantitative data were compared by the Mann-Whitney U test. All error bars represent the standard error of the mean (SEM). The presented results are representative of at least three independent biological replicates for each treatment, as indicated in the figure legends.

### Accession numbers

ERK: XM019691642 (*Ae*. *albopictus* C6/36), AALF011525 (*Ae*. *Albopictus* Foshan), AAEL013939 (*Ae*. *aegypti*), CPIJ005303 (*Cx*. *quinquefasciatus*), AGAP009207 (*An*. *gambiae*), CG12559 (*Dm*. *melanogaster*); JNK: XM019705989 (*Ae*. *albopictus* C6/36), AALF011885 (*Ae*. *albopictus* Foshan), AAEL008634 (*Ae*. *aegypti*), CPIJ001156 (*Cx*. *quinquefasciatus*), AGAP009460 (*An*. *gambiae*), CG5680 (*Dm*. *melanogaster*); p38: XM019707470 (*Ae*. *albopictus* C6/36), AALF014535(*Ae*. *albopictus* Foshan), AAEL008379 (*Ae*. *aegypti*), CPIJ002174 (*Cx*. *quinquefasciatus*), AGAP012148 (*An*. *gambiae*), CG33338 (*Dm*. *melanogaster*). The other accession numbers are shown in S2 Table.

## Results

### Different roles of MAPKs in DENV-infected *Aedes* mosquito cells

Previous studies have suggested that MAPKs regulate a variety of cellular processes, including growth, metabolism and apoptosis [23, 27, 28], and contribute to the host defense against pathogen infection in *Drosophila* [31, 33, 34]. Through a combination of the KEGG pathway and BLAST analyses, we identified three canonical components of MAPKs (ERK, JNK and p38) in both *Ae*. *aegypti* and *Ae*. *albopictus*. Further analysis of their evolutionary relationships showed that the MAPKs derived from *Aedes* mosquitoes exhibited high homology to those from *An*. *gambiae*, *Cx*. *quinquefasciatus* and *Dm*. *melanogaster* (S1 Fig).

**Fig 1:**
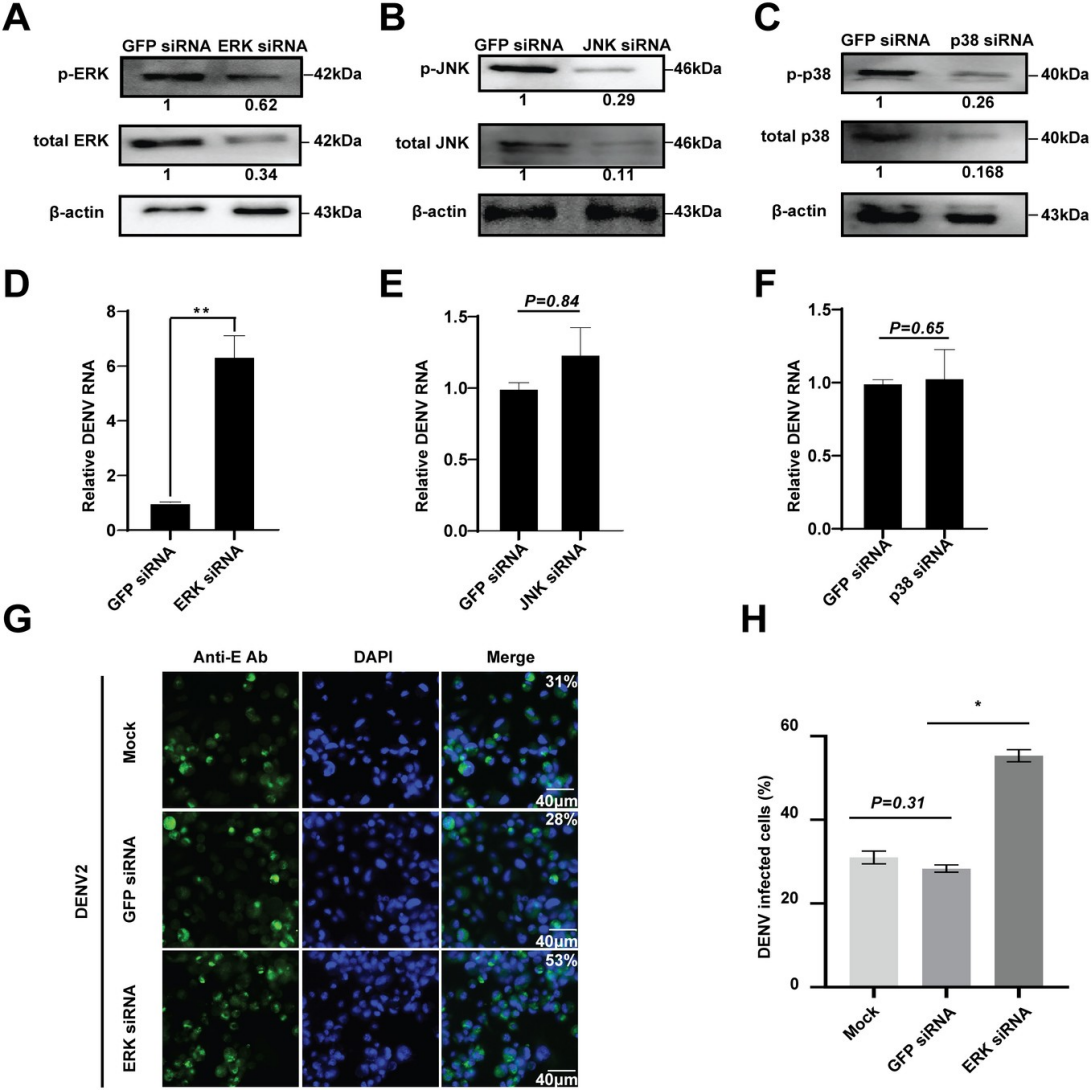
Function of MAPKs in *Aedes* mosquito cells following DENV infection. **A-C**, Knockdown of 3 MAPKs in C6/36 cells. After transfection of the indicated siRNA in C6/36 cells (**A**, ERK; **B**, JNK; **C**, p38), the levels of phosphorylated and total proteins of ERK, JNK and p38 were examined by Western blotting. β-actin was used to normalize the levels of phosphorylated and total MAPK proteins by using densitometric analyses. The fold changes were indicated under the phosphorylated and total MAPK bands relative to the GFP siRNA baselines. **D-F**, Effect of MAPKs knockdown on the viral replication in DENV2 infected C6/36 cells (**D**, ERK; **E**, JNK; **F**, p38), GFP siRNA transfection served as the control. The relative DENV mRNA level was measured using the comparative C(T) method [45]. Error bars represent SEM from three independent experiments (** *P* < 0.01; t-test). **G** and **H**, Knockdown of ERK increases the DENV titers in C6/36 cells. The viral infected cells after ERK knockdown were detected by IFA analysis, using an anti-DENV2 E monoclonal antibody (**G**). Quantification of images in **G** were generated from three independent experiments (**H**). The Mock and GFP siRNA transfection served as the controls.* *P* < 0.05; t-test.

To investigate the role of MAPKs in *Aedes* mosquitoes following DENV infection, we silenced the *ERK*, *JNK*, and *p38* genes in C6/36 cells by siRNA transfection, respectively. The mRNA levels of ERK, JNK and p38 were significantly down-regulated (S2 Fig), which was accompanied by decrease levels of their phosphorylated and total proteins, compared to the GFP siRNA control (Fig 1A–1C). We then measured the viral replication in C6/36 cells after knockdown of 3 MAPKs, respectively. The results showed that knockdown of ERK significantly enhanced the viral mRNA level by more than 6-fold (Fig 1D), and increased the number of DENV2 infected cells by around 2-fold, compared to the controls (Fig 1G and 1H). However, knockdown of either JNK or p38 had little impact on the viral replication in the DENV2 infected C6/36 cells (Fig 1E and 1F). These results suggested that ERK, but not JNK or p38, plays a role in restricting DENV infection in *Aedes* mosquitoes.

### DENV infection activates the Ras/ERK pathway of *Aedes* mosquitoes

**Fig 2:**
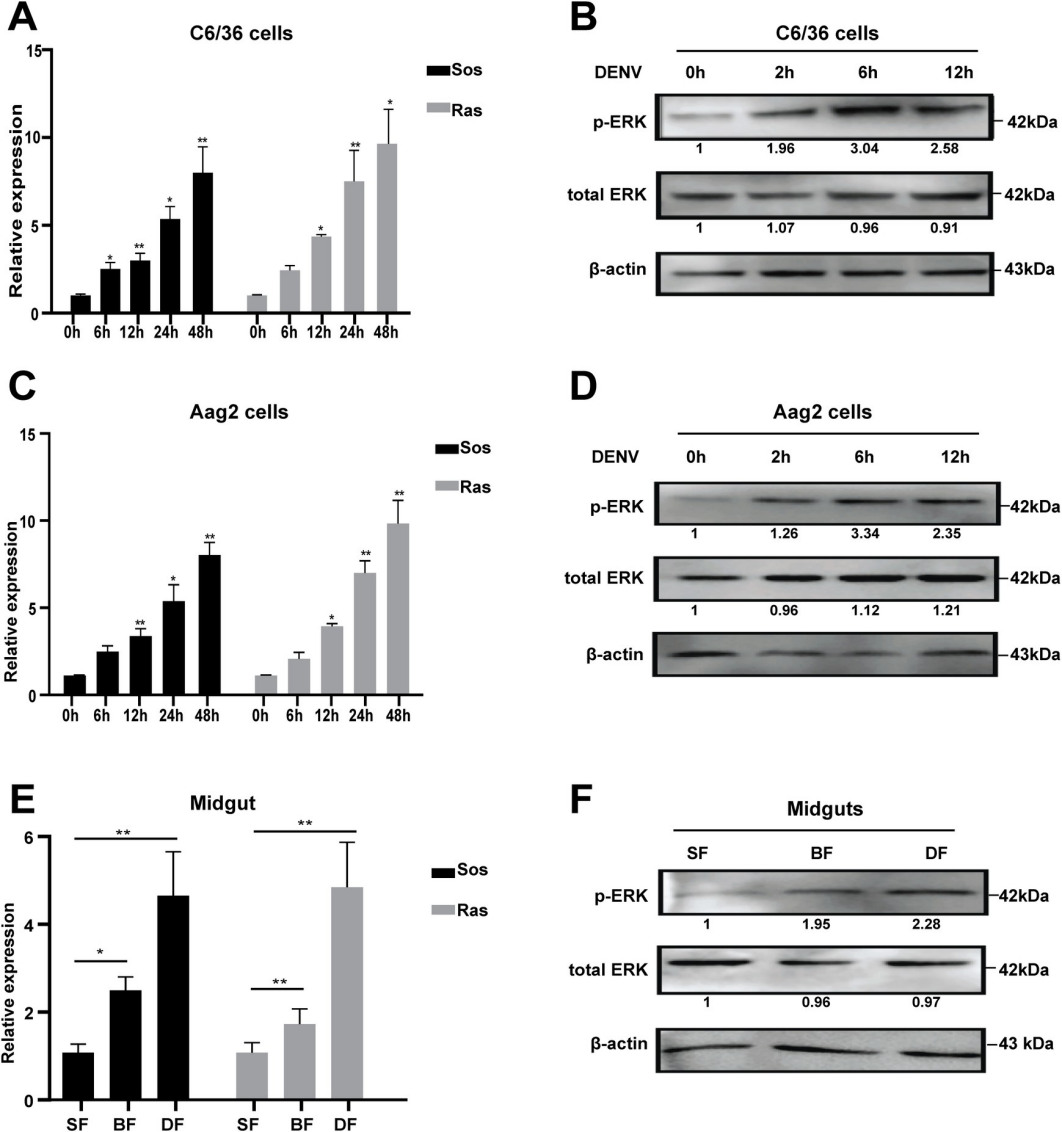
DENV activates the Ras/ERK pathway in *Aedes* mosquitoes. **A**-**D,** DENV2 infection activates the Ras/ERK pathway in *Aedes* mosquito cells. DENV2 induces the expression of *Sos* and *Ras* genes in C6/36 (**A**) and Aag2 cells (**C**). C6/36 and Aag2 were infected with DENV2, the RNAs at 6, 12, 24 and 48 h.p.i were extracted for qPCR assay. The uninfected cells were taken as the mock control (0 h). Error bars represent SEM from three independent experiments (* *P* < 0.05, ** *P* < 0.01; t-test). DENV2 infection increased ERK phosphorylation in C6/36 (**B)** and Aag2 (**D**). C6/36 and Aag2 were infected with DENV2, the proteins at 2, 6 and 12 h.p.i. were submitted for Western blotting analysis. The fold changes were indicated under the phosphorylated and total ERK bands relative to the 0 h baselines. **E** and **F**, DENV2 induces the expression of *Sos* and *Ras* genes (**E**), as well as ERK phosphorylation (**F**) in mosquito midguts. SF indicates sugar-feeding midguts; BF indicates blood-feeding midguts; DF indicates DENV2-infected midguts. * *P* < 0.05, ** *P* < 0.01; t-test.

The core components of the Ras/ERK signaling cascade include Sos, Ras, and three MAPKs: RAF (MAPKKK), MEK (MAPKK) and ERK (MAPK) [46]. By performing sequence alignment and phylogenetic analyses, we confirmed that Sos, Ras, RAF, MEK, and ERK are highly conserved in *Ae*. *albopictus*, *Ae*. *aegypti*, *An*. *gambiae*, *Cx*. *quinquefasciatus* and *Dm*. *melanogaster* (S3 Fig). To examine the response pattern of Ras/ERK signaling of *Aedes* mosquitoes in the early stage of DENV infection, we analyzed two cell lines derived from *Aedes* mosquitoes: C6/36 and Aag2. DENV2 infection induced the up-regulation of both *Sos* and *Ras* genes from 6 to 48 h.p.i. in both C6/36 and Aag2 cells, compared to those observed in the mock controls (0 h) (Fig 2A and 2C). In addition, the phosphorylated levels of ERK protein in *Aedes* mosquito cells increased from 2 h to 12 h following DENV2 infection (Fig 2B and 2D), suggesting that DENV2 infection activates the Ras/ERK pathway in *Aedes* mosquito cells.

Consistent with these observation in the cells, the up-regulation of both *Sos* and *Ras* genes, as well as the enhanced ERK phosphorylation, were confirmed in the midguts after feeding the female adult of *Ae*. *albopictus* mosquitoes with DENV2-infected blood meals (Fig 2E and 2F). Furthermore, we observed that the blood-feeding midguts without virus also exhibited induced expression of *Sos* and *Ras* genes, as well as the ERK phosphorylation (Fig 2E and 2F), consistent with the results of previous studies showing that the host blood serum-derived factors can activate the ERK/MAPK pathway in *Anopheles* mosquitoes [47, 48]. Therefore, the artificial blood that only contains red blood cells (RBC) was used in the subsequent mosquito experiments to minimize the impact of blood serum-derived factors on the ERK pathway.

### Activation of the Ras/ERK pathway restricts dengue virus infection in *Aedes* mosquito cells

**Fig 3:**
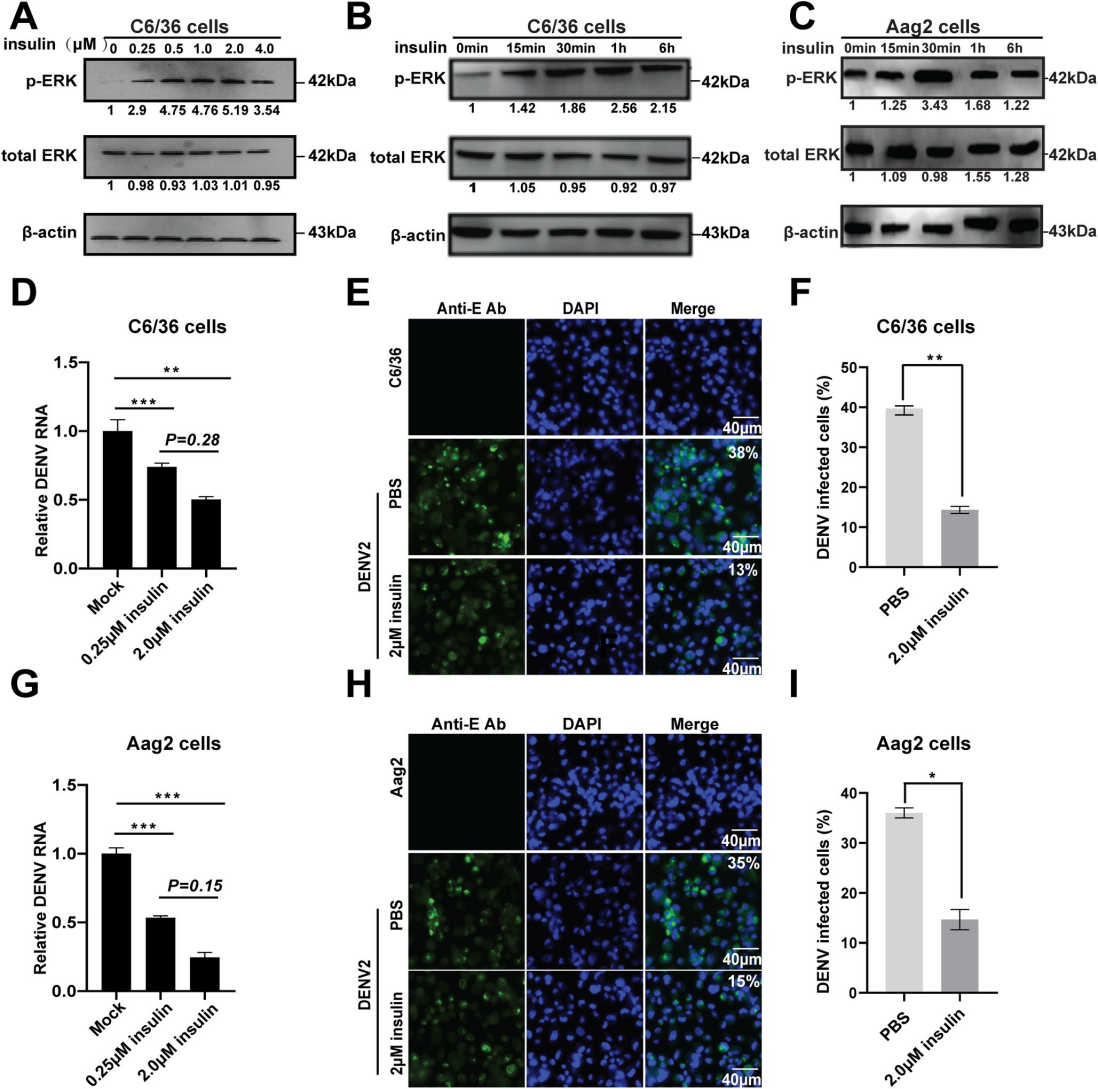
Insulin-induced ERK phosphorylation inhibits DENV replication in *Aedes* mosquito cells. **A-C**, Insulin induces ERK phosphorylation in a dose-dependently in C6/36 cells (**A**), and for the time indicated in both C6/36 (**B**) and Aag2 (**C**) cells with a dose of 2μM. The fold changes were indicated under the phosphorylated and total ERK bands relative to the control baselines. **D-F,** insulin-induced ERK phosphorylation reduced DENV titers in *Aedes* mosquito cells. **D,** C6/36 cells were pretreated with 0.25μM, 2μM insulin, or equivalent volumes of PBS for 1 h and then infected with DENV2. Total RNA was collected at 36 h.p.i., and the viral RNA levels were detected by qPCR. Error bars represent SEM from three independent experiments (** *P* < 0.01, *** *P* < 0.001; t-test). **E,** C6/36 cells were pretreated with 2μM insulin or equivalent volumes of PBS for 1 h and then infected with DENV2. Cells were fixed at 36 h.p.i., and the DENV2 infected cells were detected by IFA analysis using an anti-DENV2 E monoclonal antibody. **F**, quantification of images in **E** were generated from three independent experiments (** *P* < 0.01; t-test). The Mock cells and PBS treatment served as controls. **G-I**, Aag2 cells were treated in the same way as **D-F.** Error bars represent SEM from three independent experiments. * *P* < 0.05, *** *P* < 0.001; t-test.

To determine whether the Ras/ERK pathway is involved in the anti-dengue response in mosquito cells, we use human insulin as an agonist of ERK signaling since it promotes ERK phosphorylation in insect cells [31]. C6/36 cells were treated with different doses of human insulin (0.25–4 μM) and then analyzed for the levels of phosphorylated and total ERK. Compared to the control with PBS treatment (0 μM), ERK phosphorylation induced by insulin at a dose of 0.25 μM and increased in a dose-dependent manner, with a peak at a dose of 2 μM (Fig 3A). The levels of phosphorylated ERK could persist for at least 6 hours with 2 μM insulin treatment (Fig 3B). A similar increase in the level of ERK phosphorylation was also detected in Aag2 cells treated with 2 μM insulin (Fig 3C).

After pretreatment with insulin, C6/36 and Aag2 were separately infected with DENV2 for 36 h. We found that the insulin treatment in two doses (0.25 and 2 μM) significantly inhibited the viral mRNA replication in both C6/36 and Aag2 cells, compared to the mock controls (Fig 3D and 3G). And a high-dose insulin treatment exhibited more notably inhibitory effects on DENV2 replication, which had no significant difference relative to a low-dose treatment (Fig 3D and 3G). We further demonstrated that the insulin treatment significantly reduced the number of DENV2 infected cells, as only 13% of insulin-treated C6/36 cells were positive for DENV 2 E protein compared with the 38% of DENV 2 E positive cells observed in the PBS control by IFA analysis (Fig 3E and 3F). A similar reduction in the ratio of DENV infected cells in total number of cells was also observed in the insulin-treated Aag2 cells (Fig 3H and 3I). These results suggested that insulin-induced ERK phosphorylation inhibits DENV replication in *Aedes* mosquito cells.

**Fig 4:**
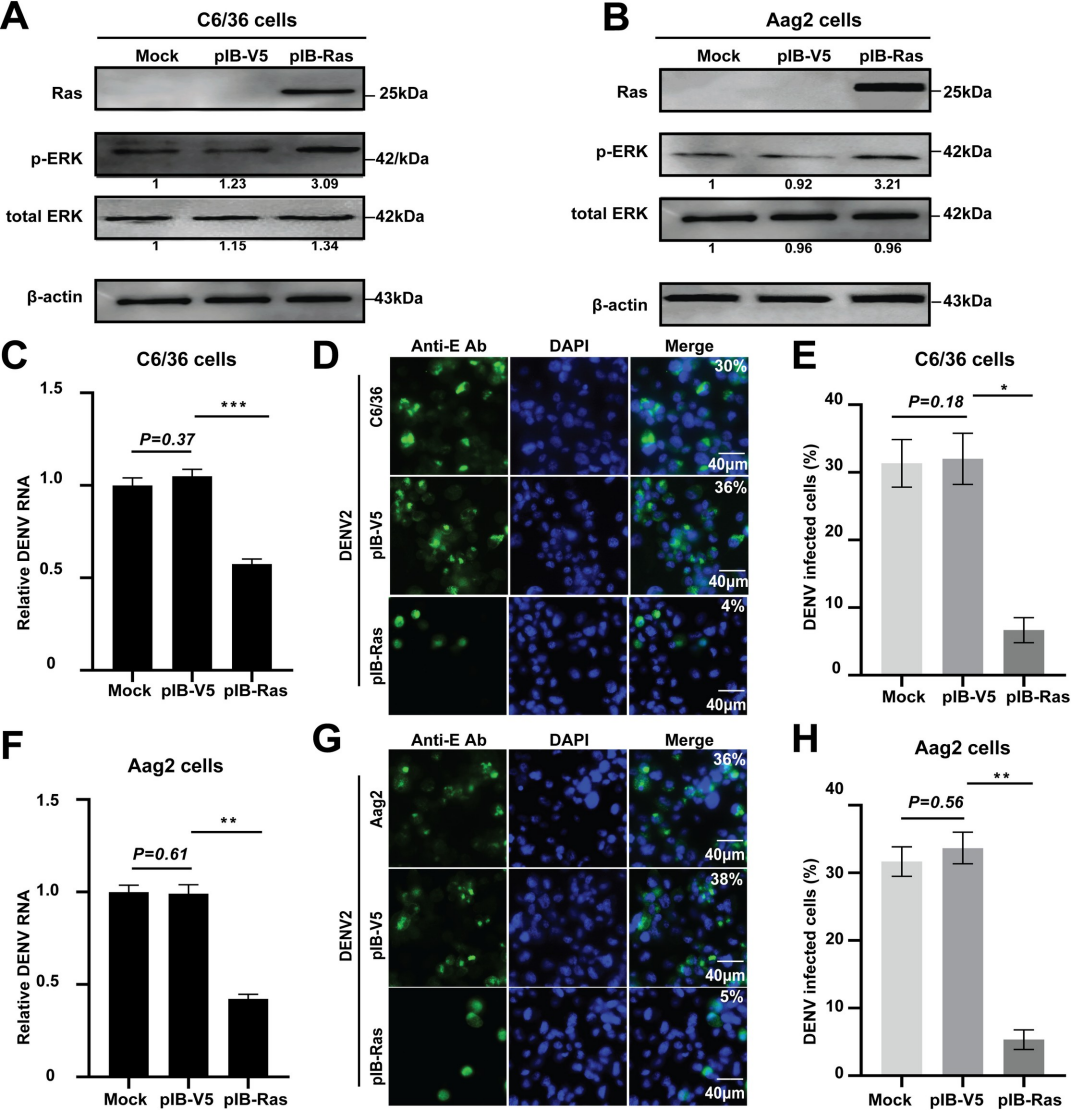
ERK phosphorylation induced by Ras overexpression reduces the DENV titers in *Aedes* mosquito cells. **A** and **B,** Ras overexpression increases ERK phosphorylation in *Aedes* mosquito cells. The recombinant plasmid pIB-Ras was separately transfected into C6/36 (A) and Aag2 (B) cells for 48 h, respectively. The proteins were extracted and subjected to SDS-PAGE and Western blotting analysis. Anti-Flag monoclonal antibody was used as the primary antibody to detect the expression of Ras. Anti-phosphorylated ERK and total ERK antibodies were used to analyze ERK activation. The fold changes were indicated under the phosphorylated and total ERK bands relative to the mock baselines. **C-H**, Ras overexpression reduces the DENV titers in *Aedes* mosquito cells. After transfection with pIB-Ras for 12 h, cells were infected with DENV2 for 36 h. The viral RNA replication was detected by qPCR (**C, F**). Error bars represent SEM from three independent experiments, ** *P*< 0.01, *** *P* < 0.001; t-test. The number of DENV2 infected cells was analyzed by IFA (**D, G**). **E** and **H**, quantification of images in **D** and **G** generated from three independent experiments. The mock and pIB-V5 vector transfected cells were used as the controls. * *P*< 0.05, ** *P*< 0.01; t-test.

Considering Ras is the initiating factor of ERK phosphorylation in the cytoplasm, we next sought to examine whether the genetic activation of ERK signaling can generate a similar inhibitory effect on DENV replication to that of the insulin treatment. According to the amino acid sequence alignment, we found that *Aedes* mosquitoes Ras has 100% identity in both *Ae*. *albopictus* and *Ae*. *aegypti* (S3 Fig). The recombinant plasmid pIB-Ras harboring the *Ae*. *albopictus Ras* gene and a FLAG tag was transfected into C6/36 and Aag2 cells, respectively. Ras overexpression was confirmed in both C6/36 and Aag2 cells by using Western blotting with an anti-FLAG monoclonal antibody (Fig 4A and 4B). As expected, we demonstrated that the levels of phosphorylated ERK were increased in the Ras-overexpressed cells (Fig 4A and 4B). Following DENV2 infection, the viral mRNA replication in the Ras-overexpressed cells was significantly inhibited relative to the control cells (Fig 4C and 4F). Furthermore, the number of DENV2 infected cells was also decreased after Ras overexpression, as only 4–5% of *Ras*-overexpressed cells tested positive for viral infection compared with 36–38% for pIB-V5 vector-transfected cells (Fig 4D and 4G). These results suggested that the genetic activation of the Ras/ERK pathway by Ras overexpression reduces the DENV titers in *Aedes* mosquito cells.

### Inhibition of the Ras/ERK pathway enhances dengue virus infection in *Aedes* mosquito cells

**Fig 5:**
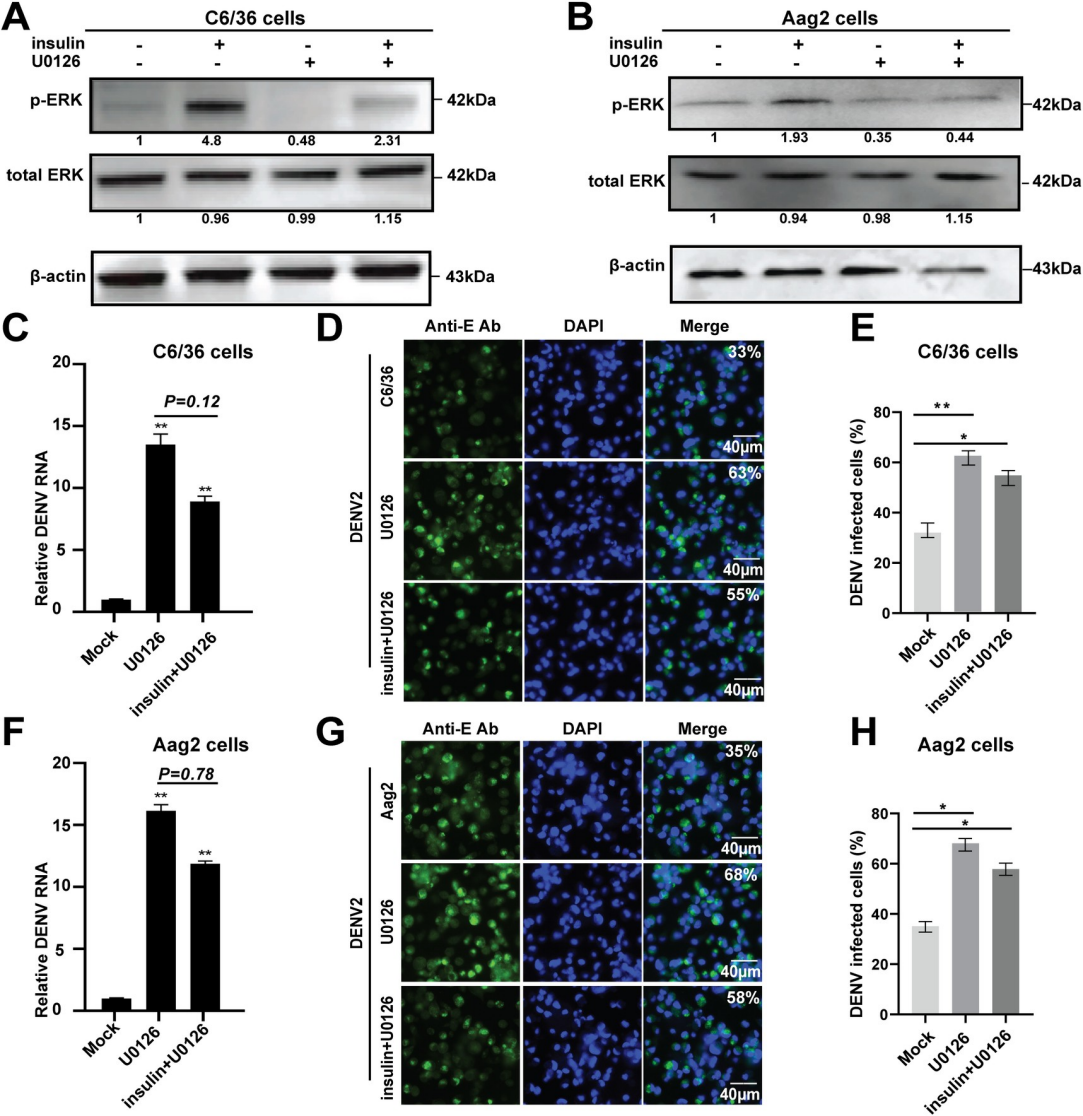
U0126 inhibition of ERK phosphorylation enhances the DENV replication in *Aedes* mosquito cells. **A** and **B**, The MEK1/2 inhibitor U0126 inhibited insulin-dependent ERK phosphorylation in C6/36 (**A**) and Aag2 cells (**B**). Cells were pretreated with U0126 alone or simultaneously with insulin for 30 min. Cell lysates were analyzed by Western blotting by using an anti-phospho-ERK or anti-total ERK antibodies. The fold changes were indicated under the phosphorylated and total ERK bands relative to the mock baselines. **C-H**, Inhibition of ERK phosphorylation by U0126 treatment enhances DENV2 replication in *Aedes* mosquito cells. After treatment with U0126 alone or simultaneous treatment with U0126 and insulin, cells were infected with DENV2 for 36 h. The DENV titers were analyzed by qPCR (**C**, C6/36 cells; **F**, Aag2 cells) and IFA (**D,** C6/36 cells; **G,** Aag2 cells). **E** and **H**, quantification of images in **D** and **G** were generated from three independent experiments. Error bars represent SEM from three independent experiments. * *P* < 0.05, ** *P* < 0.01; t-test.

Since insulin-induced ERK signaling can inhibit DENV replication in *Aedes* mosquito cells, we used U0126, a selective inhibitor of MEK across diverse species [31, 49], to inhibit the ERK signaling in *Aedes* mosquito cells. We observed that U0126 treatment decreased the levels of phosphorylated ERK and blocked the insulin-induced ERK activation in both C6/36 and Aag2 cells (Fig 5A and 5B). After DENV2 infection, the viral replication was significantly enhanced in both U0126-treated C6/36 and Aag2 cells, with an approximately10-fold increase in the viral mRNA levels, compared to the PBS-treated controls (Fig 5C and 5F). Furthermore, the number of DENV2-infected cells was also significantly increased in the U0126-treated cells (Fig 5D–5H). In addition, we found that simultaneous treatment with U0126 and insulin showed no significant difference in the viral titers compared with the U0126 treatment alone (Fig 5C and 5F), which was expected since U0126 can block the insulin-induced ERK phosphorylation, thereby neutralizing the insulin-mediated anti-DENV activity.

**Fig 6:**
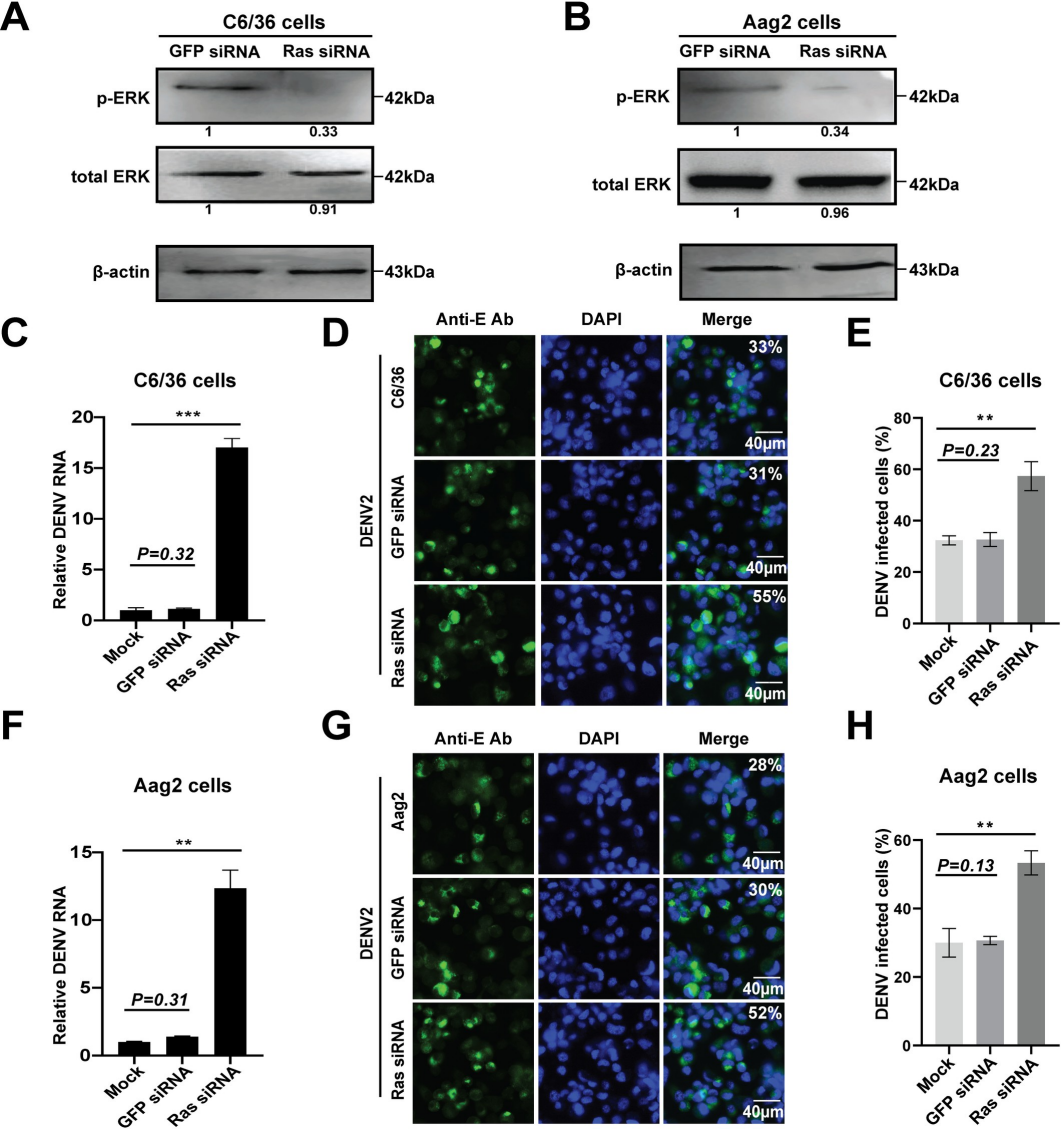
Knockdown of Ras decreases ERK phosphorylation and increases the DENV titers in *Aedes* mosquito cells. **A** and **B**, knockdown of Ras decreased ERK phosphorylation in *Aedes* mosquito cells. *Ras* siRNA oligo was transfected into C6/36 cells (**A**) and Aag2 cells (**B**), respectively. The proteins were extracted and subjected to SDS-PAGE and Western blotting analysis. Anti-phosphorylated ERK and total ERK antibodies were used to analyze ERK protein. The fold changes were indicated under the phosphorylated and total ERK bands relative to the GFP siRNA baselines. **C-H**, DENV2 titers is increased in the *Ras* knockdown cells. *Aedes* mosquito cells were transfected with *Ras* siRNA for 12h, and then infected with DENV for 36 h. Viral RNA replication and viral E protein yield were detected by qPCR (**C**, C6/36 cells; **F**, Aag2 cells) and IFA (**D,** C6/36 cells; **G,** Aag2 cells). **E** and **H**, quantification of images in **D** and **G** were generated from three independent experiments. *GFP* siRNA was used as the control. Error bars represent SEM from three independent experiments. ** *P* < 0.01, *** *P* < 0.001; t-test.

Next, we transfected *Ras* siRNA oligos into *Aedes* mosquito cells (S4 Fig). Knockdown of *Ras* resulted in a significant reduction in the level of phosphorylated ERK without altering the total ERK level (Fig 6A and 6B), suggesting that the Ras/ERK pathway was endogenously blocked. The DENV2 infected titers were then analyzed in the Ras-knockdown cells. We observed a significant increase in both the viral mRNA replication and the number of DENV2 -infected cells in the *Ras* siRNA treatment, compared to the *GFP* siRNA control (Fig 6C–6H). These results indicated that the inhibition of the Ras/ERK signaling pathway, either by U0126 treatment or by transfection of *Ras* siRNA, enhanced DENV infection in *Aedes* mosquito cells.

### Signaling crosstalk between the Ras/ERK pathway and DENV-induced AMPs

**Fig 7:**
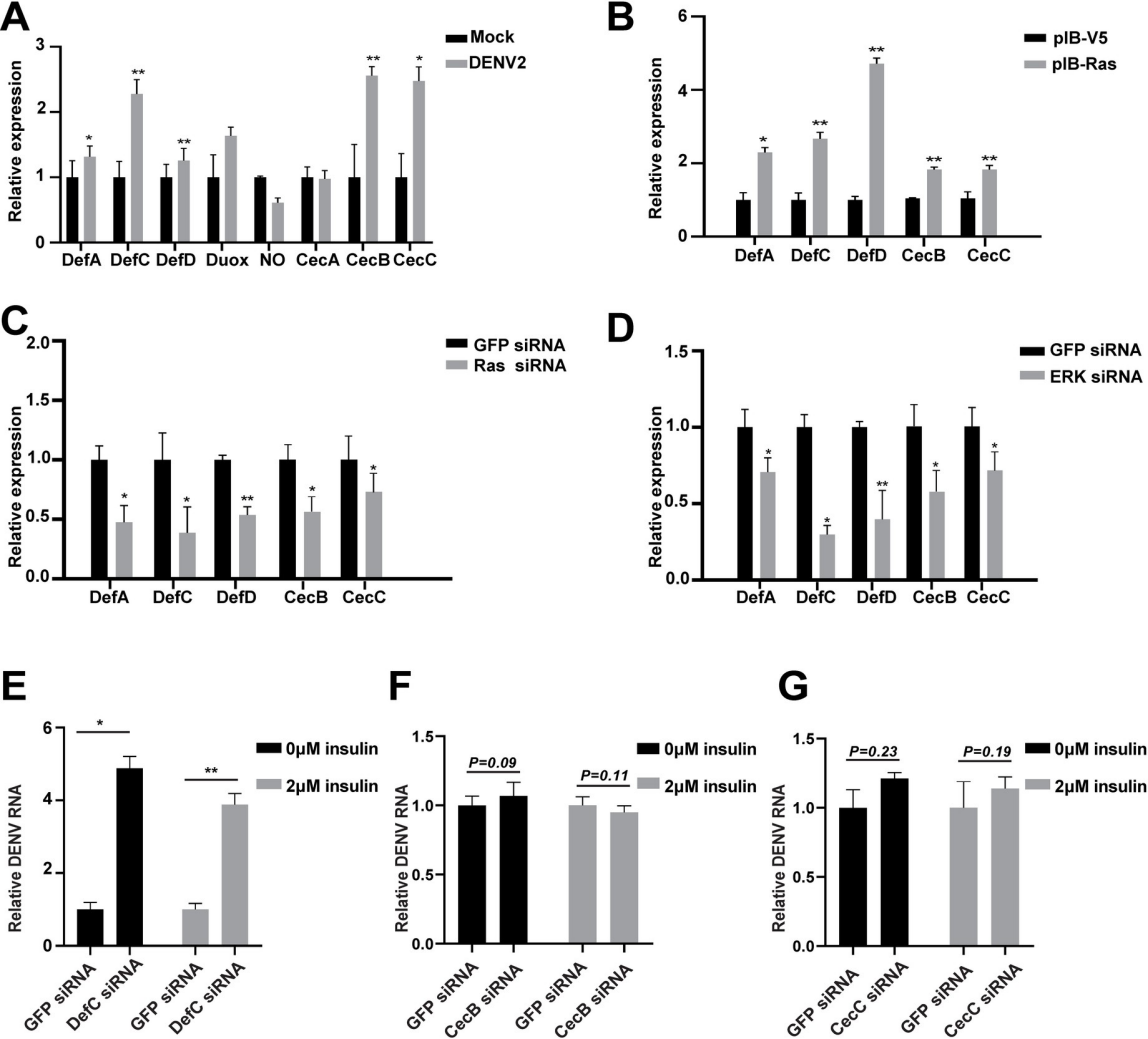
Crosstalk between the Ras/ERK pathway and DENV-induced AMPs. **A**, DENV2 induces the expression of *AMP* genes in C6/36 cells. The mRNA levels of 6 *AMPs*: *Def A*, -*C*, -*D*, *Cec A*, -*B*, and -*C*, as well as *Duox* and *Nos* genes were examined in C6/36 cells after DENV infection for 24h. The uninfected C6/36 cells were taken as the Mock control. **B**, *Ras* overexpression increases the expression of *AMP*s in C6/36 cells. C6/36 cells were transfected with pIB-Ras for 48h, the pIB-V5 vector was taken as the control. The total RNAs were extracted and the *AMPs* transcription was analyzed by qPCR. **C** and **D**, knockdown of *Ras* and *ERK* decreases *AMPs* expression in C6/36 cells. Transfection of siRNA oligo for *Ras* (**C**) and *ERK* (**D**) respectively. The total RNAs were extracted and the AMPs transcription was analyzed by qPCR. GFP siRNA was taken as the control. Error bars represent SEM from three independent experiments, * *P* < 0.05, ** *P* < 0.01; t-test. **E-G,** Effect on DENV replication after *AMPs* knockdown in C6/36 cells without or with insulin treatment. After the *AMPs* siRNA transfection, the C6/36 cells were pre-treated with insulin and then infected with DENV2 for 36 h, and the viral titers was assessed via qPCR. *GFP* siRNA was taken as the control. Error bars represent SEM from three independent experiments.* *P* < 0.05, ** *P* < 0.01; t-test.

Insects produce several effector factors such as AMPs and reactive oxygen species (ROS) to control the pathogens infection [15, 18, 20]. To investigate whether there is an association between the Ras/ERK pathway and AMPs/ROS, we selected eight candidate factors that have been indicated to be involved in the innate immunity of *Aedes* mosquitoes, including defensins (Def) and cecropins (Cec) AMP families [18, 20], Dual oxidase (Duox) and NO synthase (Nos) [30, 50]. First, the expression levels of *AMP* and *ROS* genes in C6/36 cells following DENV infection were examined. The expression of 5 *AMP*s genes, including *Def A*, *Def C*, *Def D*, *Cec B*, and *Cec C* were induced by DENV2 infection (Fig 7A). We next assessed the association between the Ras/ERK pathway and the DENV-induced *AMP*s. Activation of the Ras/ERK pathway by Ras overexpression increased the expression levels of 3 *Defs* (*Def A*, *C*, and *D*) and 2 *Cecs* (*Cec B* and *C*) in C6/36 cells (Fig 7B). In contrast, blocking of the Ras/ERK pathway significantly decreased the expression of above 5 *AMP*s by applying siRNAs to separately silence *Ras* and *ERK* genes (Fig 7C and 7D), suggesting a crosstalk between the Ras/ERK pathway and DENV-induced *AMP*s in *Aedes* mosquito cells.

Because some of the above *AMP* genes have been previously shown to be modulated by other pathways, especially the Toll pathway[18], we next investigated whether the association between the Ras/ERK pathway and *AMPs* expression is connected by the Toll pathway. Inhibition of the Ras/ERK pathway by using *Ras* siRNA showed no effect on the mRNA transcription of Rel1, a core component of the Toll pathway (S5A Fig). Similarly, blocking the Toll pathway by applying Rel1 siRNA has no impact on the expression of *Ras* gene (S5B Fig). Previous studies indicated that the increased level of ROS resulted in activation of the Toll pathway in *Ae*. *aegypti* mosquitoes [50]. However, we did not observe any significant change in the expression level of either *Doux* or *Nos* gene after activation or inhibition of the Ras/ERK pathway in C6/36 cells (S6 Fig). These results indicated that the crosstalk between the Ras/ERK pathway and *AMP*s expression might be independent of the Toll pathway.

To further elucidate the antiviral roles of the above *AMP*s, we silenced 3 *AMP* genes: *Cec B*, *Cec C* and *Def C* (S7 Fig), and then assessed the effect on viral replication in C6/36 cells post-DENV2 infection. Our results showed that knockdown of *Def C* significantly enhanced the viral RNA replication without insulin treatment (0 μM) and with 2 μM insulin treatment (Fig 7E). However, we did not observe any effect on the viral RNA replication after knockdown of either *Cec B* or *Cec C* (Fig 7F and 7G), indicating that *Def C*, rather than *Cec B* or *Cec C*, participated in the resistance of Ras/ERK signaling to DENV in *Aedes* mosquitoes.

### The Ras/ERK pathway coupled AMPs to resist DENV infection in the mosquito midguts

**Fig 8:**
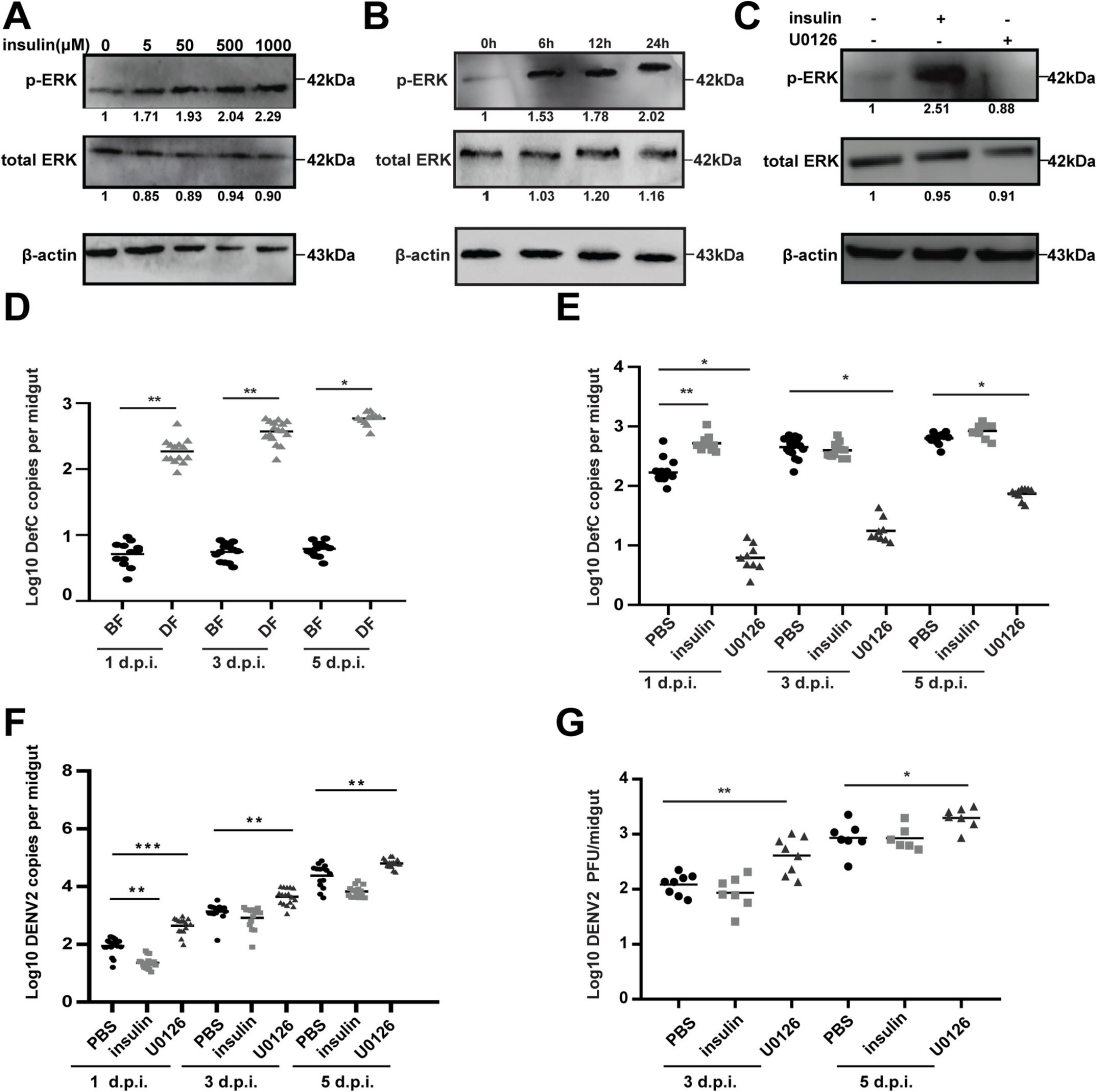
The Ras/ERK pathway couples AMPs to inhibit DENV infection in mosquito midguts. **A-C**, ERK phosphorylation in the midguts of *Ae*. *albopictus* were activated and inhibited by Insulin and U0126 priming, respectively. **A**, Feeding with artificial blood meals supplemented with 5–1000 μM insulin enhanced ERK phosphorylation level in mosquito midguts. **B**, Female adults were fed with 500 μM insulin from 6–24 h. midguts from each treatment group were dissected and prepared for Western blotting. **C**, Inhibition of ERK phosphorylation in mosquito midguts by feeding with U0126. The fold-change was indicated under the phosphorylated and total ERK bands relative to the baseline. **D,** DENV2 induces *Def C* expression in mosquito midguts. After feeding female mosquitoes with artificial blood meals supplemented DENV2 for 2 h, the midguts of blood-fed mosquitoes were collected at 1, 3 and 5 days for dissection and qPCR analyses. The artificial blood meals supplemented with PBS were taken as the controls. **E**, Insulin and U0126 treatments influences *Def C* expression in the midguts of *Ae*. *albopictus* mosquitoes. After feeding female mosquitoes with viral blood meals supplemented with insulin or U0126 for 2 hours, the midguts of blood-fed mosquitoes at 1,3 and 5 days were dissected and used for qPCR analyses. The viral blood meals supplemented with PBS were taken as the control. Dot plots represent gene copies for midgut samples. Black bars in the dot plots represent treatment medians. Each dot represents a single midgut sample. N = 16–20 samples per treatment. Mann-Whitney U test was used to determine P values for all comparisons, * *P* < 0.05, ** *P* < 0.01. **F and G**, Effect on the DENV titers in mosquito midguts by insulin and U0126 treatment. *Ae*. *albopictus* mosquitoes were fed on the viral blood meals supplemented with 500 μM insulin or 34 μM U0126. Blood-fed midguts from each treatment group were dissected and prepared for qPCR (G) and plaque assay (H). Dot plots represent the viral titers for infected midgut samples only, with P values determined via Mann-Whitney U test for all comparisons. N = 16–20 samples per treatment. * *P* < 0.05, ** *P* < 0.01, *** *P* < 0.001.

To investigate the functional role of the Ras/ERK pathway in mosquito midguts after taking the viral blood meals, we fed the female adult of *Ae*. *albopictus* mosquitoes with the artificial blood meals supplemented with insulin or U0126. Similar to the cells treated with insulin, a dose-dependent increase of ERK phosphorylation was observed in the mosquito midguts fed with 5–1000 μM insulin (Fig 8A). The phosphorylated ERK levels was increased in the midguts after feeding with 500 μM insulin from 6–24 hours (Fig 8B), and was inhibited by feeding with 34 μM U0126 (Fig 8C).

To determine if the Ras/ERK pathway and AMPs co-mediate the resistance to DENV infection in the mosquito midguts, the expression of AMPs in mosquito midguts was examined after feeding the viral blood meals supplemented with insulin or U0126. As shown in Fig 8D, the expression level of *Def C* was significantly up-regulated in the DENV2 infected midguts corresponding to the increased viral RNA levels at 1, 3 and 5 days post infection (d.p.i) (S8A Fig). Moreover, we observed that DENV induced *Cec C* expression at 3 and 5 d.p.i, but not for other 3 *AMP* genes (S8B–S8E Fig).

Furthermore, we demonstrated a significant up-regulation of the *Def C* expression and a decrease of the viral RNA replication in the insulin-fed midguts by 1 day post infection (d.p.i), compared to the PBS-fed controls (Fig 8E and 8F). In contrast, U0126 treatment significantly inhibited *Def C* expression and elevated DENV RNA replication in the midguts at 1, 3 and 5 d.p.i (Fig 8E and 8F). Although the insulin-induced inhibition on the viral replication did not persist for over 3 days, we observed that U0126 treatment increased the viral titers in the midguts both at 3 and 5 d.p.i. by plaque assays (Fig 8F and 8G). In addition, we did not observe any significant changes in the expression of *Cec-B* and **-***C* in either insulin- or U0126-fed midguts (S8F and S8G Fig). Collectively, these results indicate that the Ras/ERK pathway couples the expression of AMPs especially Def C to inhibit dengue virus infection in the midgut of *Ae*. *albopictus* mosquitoes.

## Discussion

The innate immune system provides the first line of defense for multicellular organisms against pathogen infections. The PRR-dependent activation of signaling pathways, including Toll, Imd, JAK-STAT and MAPKs, are crucial for generating innate immune responses in both insects and mammals [14, 23, 51]. In this study, we identified the evolutionary conservation of MAPKs in *Aedes*, *Anopheles* and *Culex* mosquitoes, as well as *Drosophila*. We demonstrated that knockdown of ERK significantly increases the DENV titers in *Aedes* mosquito cells. However, knockdown of the other two MAPKs, JNK and p38, did not alter the viral titers in the DENV-infected cells. Additional functional studies in the cells and midguts of *Aedes* mosquitoes demonstrated that the Ras/ERK signaling plays a role in controlling DENV infection, as both genetic and pharmacological activation of the Ras/ ERK signaling significantly restrict virus replication. In contrast, inhibition of the Ras/ERK signaling enhances DENV infection. Our results are consistent with previous findings in both *Drosophila* and mosquitoes cells [31, 52]. Knockdown of *ERK* in both *Drosophila* and Aag2 cells significantly enhanced the infections by several arboviruses, including vesicular stomatitis virus (VSV), Sindbis virus (SINV) and *Drosophila* C virus (DCV) [31]. In addition, activation of ERK signaling in both *Drosophila* and *Culex* cells has been shown to inhibit West Nile virus (WNV) replication [52]. The results of our studies add to growing evidence that the ERK/MAPK pathway plays an important role in mediating the resistance of *Aedes* mosquitoes to DENV2 infection, suggesting that the anti-virus function of ERK/MAPK is conserved across the insect species.

The midgut is a pivotal natural entry site for mosquito-transmitted pathogens, such as malaria and arboviruses, and provides the first-line barrier to efficiently limit pathogen infection [15, 51]. The ERK/MAPK pathway relays the signals transduced by the membrane receptor tyrosine kinases (RTKs), such as insulin receptor (InR) and epidermal growth factor receptor (EGFR), which are closely associated with the epithelial homeostasis and regeneration of the insect midgut [27, 29]. The nutrient status and symbiotic microbiota can regulate the ERK/MAPK pathway in insect guts [31, 32, 53], thereby getting involved in the innate immune response to parasites and viral infection [40, 41, 52].

In this study, we confirmed insulin treatment can induce the ERK phosphorylation, and enhance the resistance to DENV2 infection in both the cells and midguts of *Aedes* mosquitoes. However, the viral RNA was only significantly suppressed in the insulin-fed midguts at 1 d.p.i. but not after (Fig 8F). This is even more evident when the infectious virus titers were measured by plaque assays (Fig 8G), suggesting that the effect of in vivo ERK activation on the DENV2 seems to be less strong than in vitro. This possibly is due to the following reasons: a) The effect of ERK activation by insulin in vivo is transient and can’t persist for longer than 1–2 days. All the in vitro experiments were also performed at early time points so we don’t know the in vitro long term effect in this study. However, the previous study suggest against this because the effect of insulin on flavivirus restriction can persist even at five days after infection [52]. b) There is a limiting threshold that ERK can be activated in vivo, and that the DENV infection already activated ERK to that threshold on later timepoints. c)The effect of ERK pathway on DENV2 restriction is not very strong or not sufficient to overcome DENV2 once the virus can establish the infection. Because the effect of ERK activation on DENV2 replication in *Ae*. *albopictus* observed in this study was quite different from the WNV infection in *Cx*. *quinquefasciatus*. It would be important to address the differences between this current and previous studies.

Host blood-derived factors or their metabolites may affect the susceptibility of mosquitoes to parasites and arboviruses infection [47, 54]. Previous studies reveals that infection with malaria parasites can increase blood insulin levels by over10-fold than that of a healthy adult [55]. Activation of ERK signaling by blood factors will favor malaria parasites development in the midguts of *Anopheles* mosquitoes [41]. A recent report indicated that *Ae*. *aegypti* mosquitoes feeding on sideropenic mice exhibited a higher prevalence of dengue virus, suggesting that the deficiency of serum iron in the human population may contribute to and facilitate the spread of DENV by mosquitoes [54]. In our study, the dose-dependent phosphorylation of ERK were observed in the insulin treated both cells and midguts of *Aedes* mosquitoes. Moreover, a high-dose insulin treatment in vitro exhibited more notably inhibitory effects on DENV2 than that of the low dose insulin treatment. In the *Culex* mosquitoes, feeding blood with a normal level of insulin (170 pM) reduces WNV replication [52]. Our studies in vivo showed that feeding *Ae*. *albopictus* mosquitoes with a much higher level of insulin (more than 5 μM) can induce the phosphorylation of ERK, and enhance the resistance to DENV2 at the earlier infection stage. These results suggest that mosquitoes fed with flavivirus-infected human blood containing a normal or high level of insulin (hyperinsulinemia in diabetics) may enhance the mosquitoes resistance to virus infection, likely decreasing the ability of mosquitoes to transmit arboviruses. In addition, there are compelling epidemiological evidence that diabetes mellitus increases the risk for many infectious diseases, including WNV and DENV infection [56–58]. It will be useful to investigate whether the permissiveness of *Aedes* mosquitoes to DENV is correlated with the occurrence of hypoinsulinemia diabetes in dengue epidemic areas.

Multiple studies have reported that AMPs and ROS represent an important class of immune effector molecules in insect gut immunity [18, 20, 30, 50, 51]. In addition to the canonical Toll and IMD pathways, the induction of AMPs in response to infection is also regulated by the complement-like system and insulin/insulin-like growth factor signaling (IIS) in insects [20, 59]. We demonstrated a signaling crosstalk between the Ras/ERK pathway and the DENV-induced *AMP* genes, including *Def A*, *Def C*, *Def D*, *Cec B* and *Cec C*. Activation or Inhibition of the Ras/ERK pathway significantly increased or decreased the expression of the above *AMP*s. We further confirmed that knockdown of *Def C* significantly enhances the viral titers in C6/36 cells, which is consistent with the previous studies in *Ae*. *aegypti* mosquitoes, as the knockdown of *Def C* was shown to enhance the DENV titers in *Ae*. *aegypti* mosquitoes [20]. Furthermore, our results demonstrated that the Ras/ERK pathway plays a role in controlling DENV infection by coupling to *AMPs* expression which might be independent of the Toll pathway. Interestingly, a recent study indicated that activation of ERK signaling increased the expression of upd2/3, which enhanced JAK/STAT signaling to control WNV replication in *Drosophila* cells and *Culex* mosquitoes [52]. It is reasonable that multiple signaling pathway in innate immune system co-manage the systemic antiviral responses in mosquitoes [51].

Genetically engineered mosquito strains that overexpress immune effectors in the midgut and fat body have successfully reduced vector competence for parasite and viral infection [9, 12, 60]. For example, transgenic *Anopheles* mosquitoes overexpressing Relish2, the transcriptional activator of the IMD pathway, in the midgut and fat body effectively inhibited *P*. *falciparum* development [61]. A similar transgenic strategy involving activation of Akt signaling in the midgut has been used to enhance the resistance of female *An*. *stephensi* to *Plasmodium* infection [12, 60]. In *Ae*. *aegypti* mosquitoes, overexpressing the antiviral JAK-STAT pathway’s receptor Dome or signal transducer Hop has been shown to effectively inhibit DENV infection in the midgut and the salivary glands [9]. In this study, we confirmed that overexpression of Ras could endogenously activate the ERK/MAPK pathway and inhibit DENV replication in *Aedes* mosquito cells. Furthermore, we demonstrated that the Ras/ERK pathway couples AMPs to mediate the resistance to DENV in *Aedes* mosquitoes. In future, we will explore whether the genetic activation of ERK pathway in the midgut of *Aedes* mosquitoes by overexpression of pathway’s transducer such as Ras can control the transmission of DENV and other arboviruses.

## Supporting information

S1 Fig Phylogenetic analysis of MAPKs in mosquitoes and *Drosophila*. Phylogenetic tree analysis of ERK, JNK and p38 MAPKs. The tree was constructed using the neighbor-joining (NJ) method in MEGA version 7. The bootstrap values of 1000 replicates (%) are indicated on the branch nodes. *Aedes albopictus* (*Ae*. *albopictus*), *Aedes aegypti* (*Ae*. *aegypti*), *Anopheles gambiae* (*An*. *gambiae*), *Culex quinquefasciatus* (*Cx*. *quinquefasciatus)* and *Drosophila melanogaster* (*Dm*. *melanogaster*) are indicated, respectively.
(TIF)

S2 Fig The efficiency of MAPKs Knockdown in C6/36 cells. siRNA oligos against the 3 MAPKs (**A**, *ERK*; **B**, JNK; **C**, p38) were transfected into *Ae*. *albopictus* C6/36 cells for 48 h, respectively. The mRNA level was detected by qPCR. GFP siRNA was taken as the control. All experiments were repeated in triplicate. Data are represented as mean ± SEM. ** *P* < 0.01.
(TIF)

S3 Fig Sequence alignment and phylogenetic analyses of the Ras/ERK signaling pathway in mosquitoes and *Drosophila*. The sequences of Sos, Ras, RAF, MEK, and ERK were aligned using Clustal X, and an unrooted phylogenetic tree was built with MEGA 7 software by using the neighbor-joining method. The bootstrap values of 1000 replicates (%) are indicated on the branch nodes. *Ae*. *albopictus* (Aal), *Ae*. *aegypti* (Aae), *An*. *gambiae* (Ag), *Cx*. *quinquefasciatus* (Cq) and *Dm*. *melanogaster* (Dm) are indicated, individually.
(PDF)

S4 Fig Knockdown of Ras by siRNA in C6/36 and Aag2 cells. siRNA oligos against *Ras* were transfected into C6/36 cells (**A**) and Aag2 cells (**B**) for 48 h, respectively. The mRNA level of Ras was detected by qPCR. GFP siRNA was taken as the control. All experiments were repeated in triplicate. Student’s t-tests were used to determine the significance of the difference between experimental and control groups. Data are represented as mean ± SEM. * *P* < 0.05, *** *P* < 0.001.
(TIF)

S5 Fig Blocking of Ras/ERK and Toll pathways by siRNA in C6/36 cells. **A**, Ras knockdown shows little effect on the expression of *Rel1*gene. *Ras* siRNA oligos were transfected into C6/36 cells for 48 h, the mRNA level of *Ras* and *Rel1* were detected by qPCR. **B**, Rel1 knockdown shows little effect on the expression of *Ras* gene. *Rel1* siRNA oligos were transfected into C6/36 cells for 48h, the mRNA level of *Rel1*and *Ras* was detected by qPCR. *GFP* siRNA was taken as the control. The experiments were repeated at least three times. Student’s t-tests were used to determine the significance of the difference between experimental and control groups. Data are represented as mean ± SEM. * *P* < 0.05.
(TIF)

S6 Fig Genetic manipulation of the Ras/ERK pathway had little effect on the expression of Doux and Nos genes A, Ras overexpression shows little effect on the mRNA transcription of *Doux* and *Nos*. After transfection of pIB-Ras into *Ae*. *albopictus* C6/36 cells for 48 h, the mRNA level of *Doux* and *Nos* was detected by qPCR. pIB-V5 transfected cells were taken as the control. **B**, Ras knockdown shows little effect on the mRNA transcription of *Doux* and *Nos*. After transfection of *Ras* siRNA oligos individually into *Ae*. *albopictus* C6/36 cells for 48 h, the mRNA level of *Doux* and *Nos* was detected by qPCR. *GFP* siRNA was taken as the control. **C**, ERK knockdown shows little effect on the mRNA transcription of *Doux* and *Nos*. After transfection of *ERK* siRNA oligos individually into *Ae*. *albopictus* C6/36 cells for 48 h, respectively, the mRNA level of *Doux* and *Nos* were detected by qPCR. *GFP* siRNA was taken as the control. The experiments were repeated at least three times. Data are represented as mean ± SEM, t-tests.
(TIF)

S7 Fig The efficiency of AMPs knockdown in C6/36 cells. **A-C**, The siRNA oligos against *Def C* (**A**), *Cec B* (**B**) and *Cec C* (**C**) were transfected into *Ae*. *albopictus* C6/36 cells, respectively. After 48 h transfection, the mRNA level of *Cec B*, *Cec C*, and *Def C* were detected by qPCR. *GFP* siRNA was taken as the control. The experiments were repeated at least three times. Student’s t-tests were used to determine the significance of the difference between experimental and control groups in cell experiments. Data are represented as mean ± SEM. * *P* < 0.05.
(TIF)

S8 Fig The expression levels of DENV mRNA and AMP genes in the midguts of *Ae*. *albopictus* mosquito post-DENV2 infection. **A,** The transcription of DENV mRNA in the midguts of *Ae*. *albopictus* mosquitoes post-DENV2 infection. Feeding the female adult mosquitoes with the artificial blood containing DENV2, and the midgut samples from blood-fed mosquitoes were collected at 12 hours,1, 3, 5 and 7days post-infection. The viral mRNA levels in the midgut of blood-feeding mosquitoes were analyzed by qPCR. **B-E,** The expression of *AMP* genes in the midguts of *Ae*. *albopictus* mosquito post-DENV2 infection. Feeding the female adult mosquitoes with the artificial blood containing DENV2, and the midgut samples from blood-fed mosquitoes were collected at 1, 3 and 5 days post-infection. The AMP mRNA levels in the midgut of blood-feeding mosquitoes were analyzed by qPCR (**B**, *Def A*; **C**, *Def D*; **D**, *Cec B*; **E**, Cec *C*). **F** and **G,** insulin and U0126 treatments show no effect on the expression of *Cec B* and *Cec C* in the mosquito midguts. After feeding female mosquitoes with viral blood meals supplemented insulin and U0126, the midguts of blood-fed mosquitoes at 1, 3 and 5 days were dissected and used for qPCR analyses (**F**, *Cec B*; **G**, *CecC*). The viral blood meals supplemented PBS were taken as the control. DENV 2 RNA level and *AMP* genes expression in the midguts were determined using an absolute quantification qPCR method and presented in log10. Dot plots represent the viral genome (**A**) and AMP gene (**B-G**) copies for midgut samples. Black bars in the dot plots represent treatment medians. Each dot represents a single midgut sample. N = 16–20 samples per treatment. Mann-Whitney U test was used to determine *P* values for all comparisons. * *P* < 0.05, ** *P* < 0.01.
(TIF)

S1 Table The siRNA sequences for Knockdown of MAPKs in *Aedes* mosquito cells. (DOCX)

S2 Table The primer sequences for real-time qPCR. (DOCX)
