# Supplementary figures and images for: The Ras/ERK signaling pathway couples antimicrobial peptides to mediate resistance to dengue virus in Aedes mosquitoes
Source: PLoS Negl Trop Dis. 2020 Aug 31;14(8):e0008660. doi: 10.1371/journal.pntd.0008660 (PMC7485967; doi:10.1371/journal.pntd.0008660)

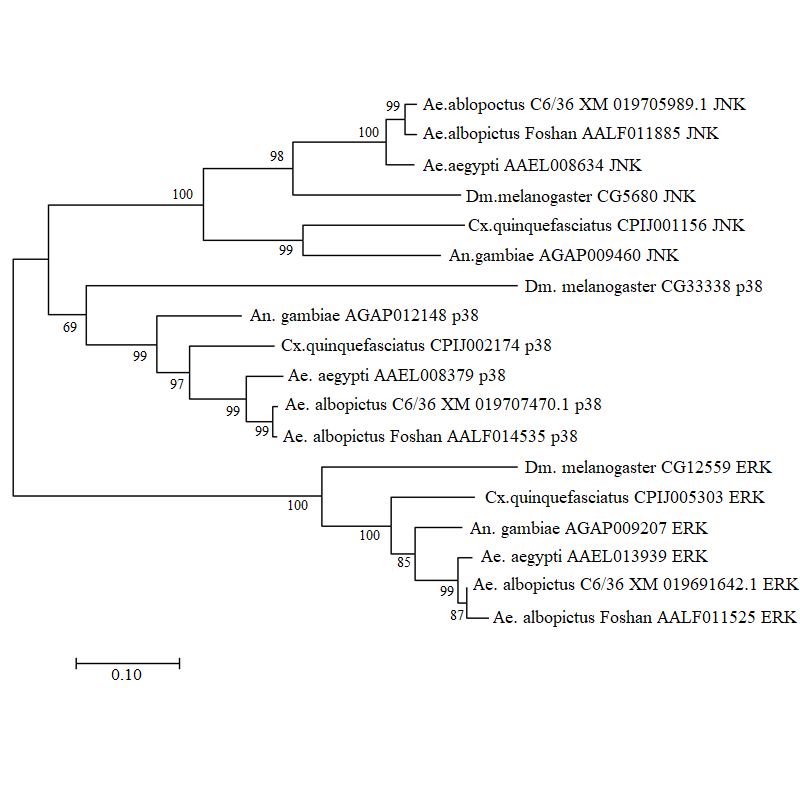

Supplement: S1 Fig — Phylogenetic tree analysis of ERK, JNK and p38 MAPKs. The tree was constructed using the neighbor-joining (NJ) method in MEGA version 7. The bootstrap values of 1000 replicates (%) are indicated on the branch nodes. Aedes albopictus (Ae. albopictus), Aedes aegypti (Ae. aegypti), Anopheles gambiae (An. gambiae), Culex quinquefasciatus (Cx. quinquefasciatus) and Drosophila melanogaster (Dm. melanogaster) are indicated, respectively. (TIF) [file pntd.0008660.s001.tif]

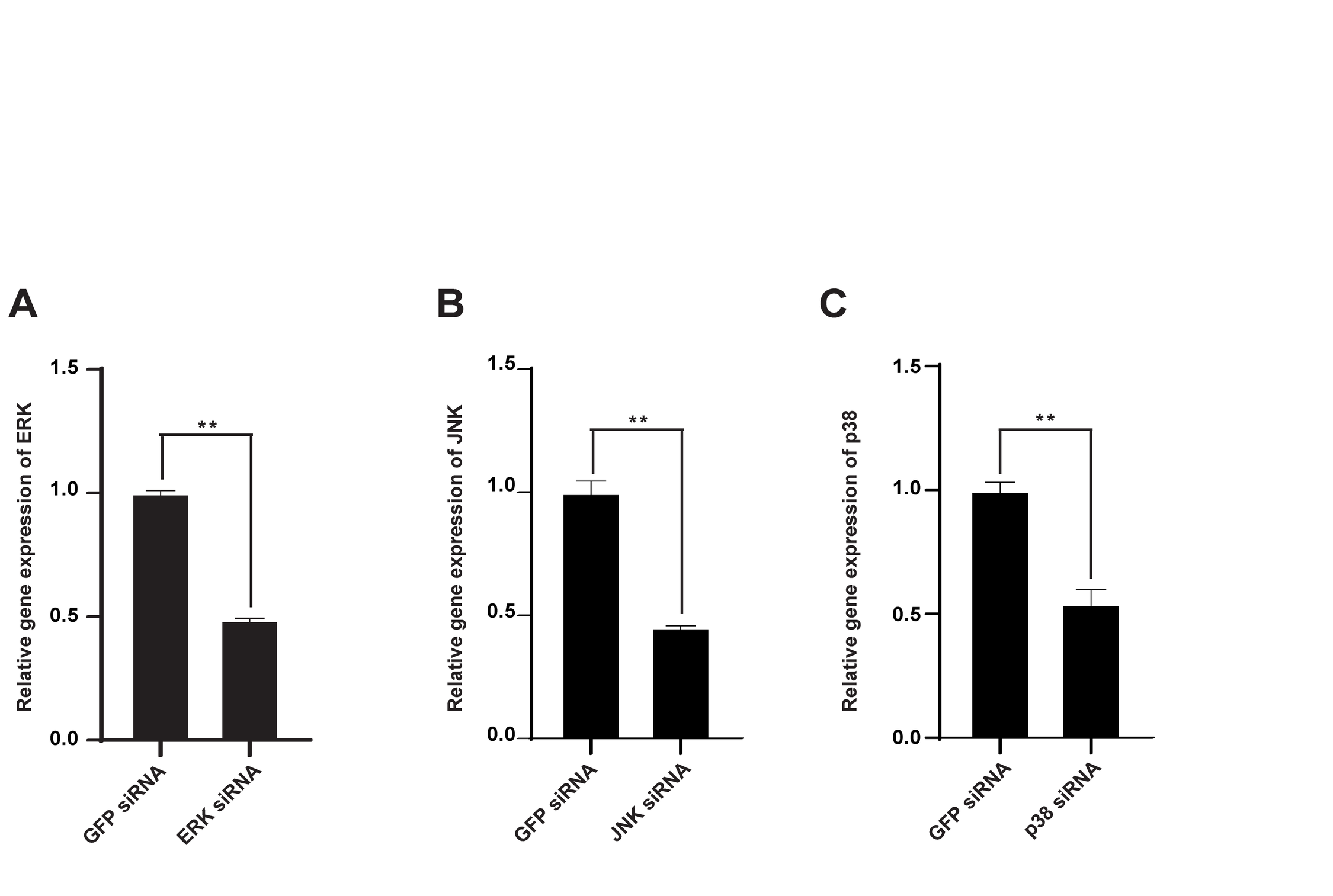

Supplement: S2 Fig — siRNA oligos against the 3 MAPKs (A, ERK; B, JNK; C, p38) were transfected into Ae. albopictus C6/36 cells for 48 h, respectively. The mRNA level was detected by qPCR. GFP siRNA was taken as the control. All experiments were repeated in triplicate. Data are represented as mean ± SEM. ** P < 0.01. (TIF) [file pntd.0008660.s002.tif]

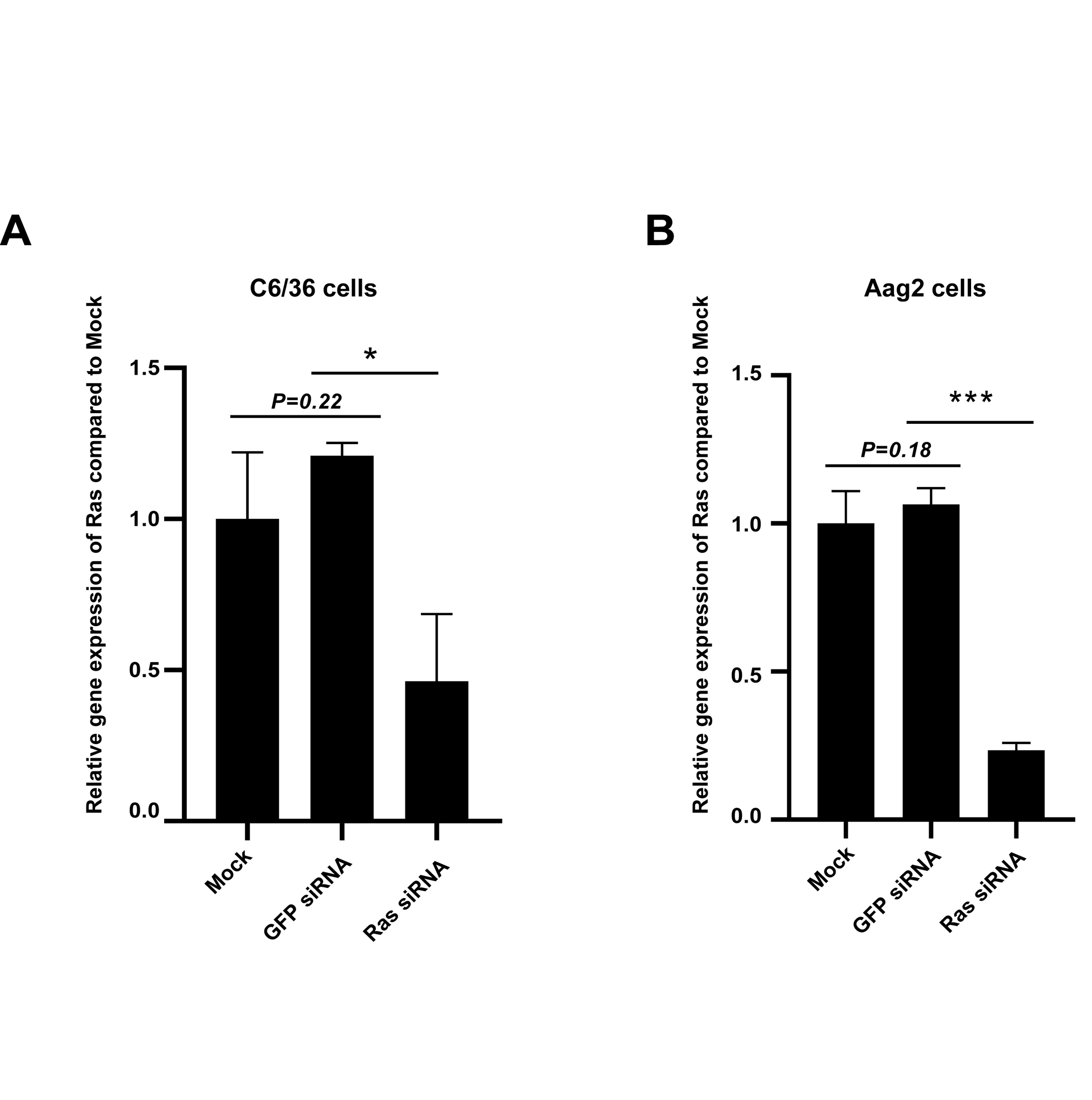

Supplement: S4 Fig — siRNA oligos against Ras were transfected into C6/36 cells (A) and Aag2 cells (B) for 48 h, respectively. The mRNA level of Ras was detected by qPCR. GFP siRNA was taken as the control. All experiments were repeated in triplicate. Student’s t-tests were used to determine the significance of the difference between experimental and control groups. Data are represented as mean ± SEM. * P < 0.05, *** P < 0.001. (TIF) [file pntd.0008660.s004.tif]

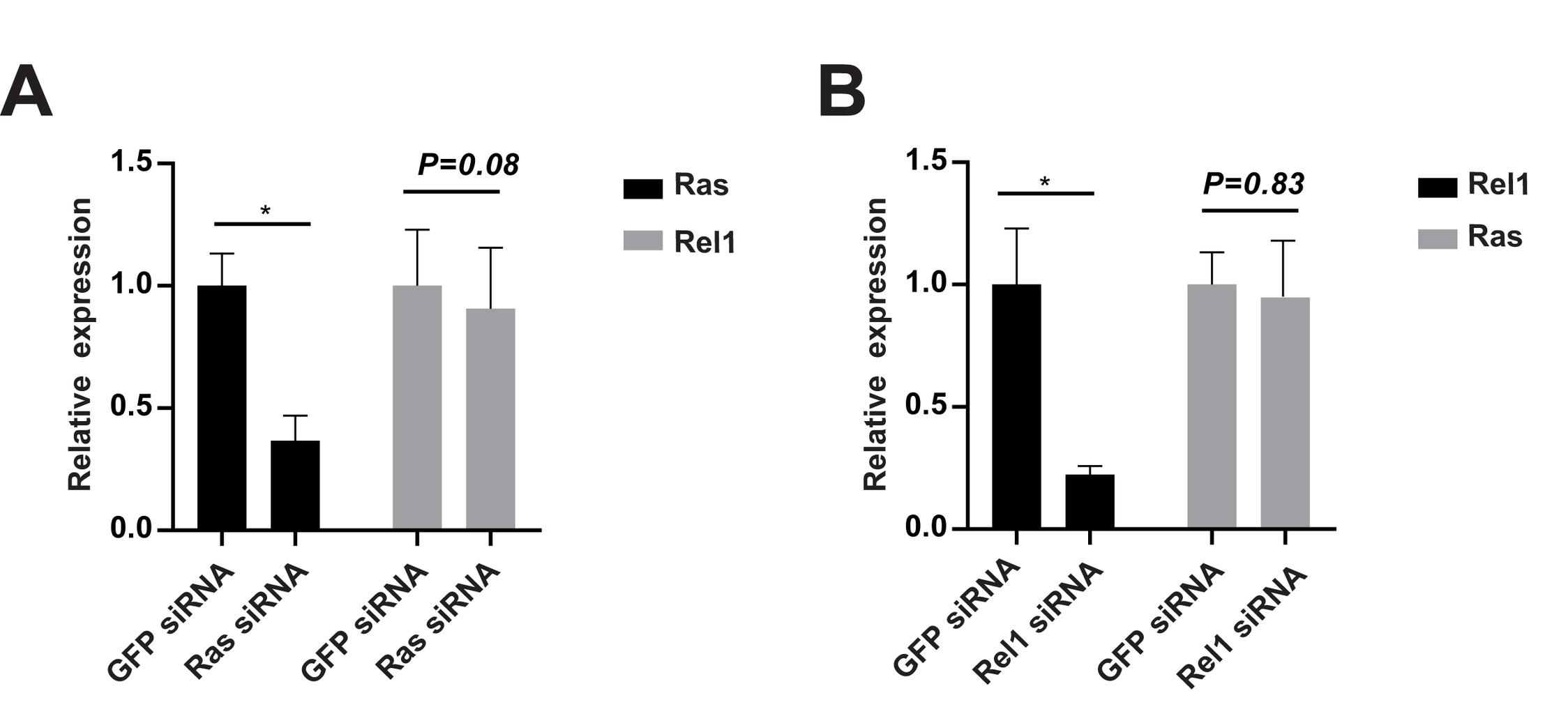

Supplement: S5 Fig — A, Ras knockdown shows little effect on the expression of Rel1gene. Ras siRNA oligos were transfected into C6/36 cells for 48 h, the mRNA level of Ras and Rel1 were detected by qPCR. B, Rel1 knockdown shows little effect on the expression of Ras gene. Rel1 siRNA oligos were transfected into C6/36 cells for 48h, the mRNA level of Rel1and Ras was detected by qPCR. GFP siRNA was taken as the control. The experiments were repeated at least three times. Student’s t-tests were used to determine the significance of the difference between experimental and control groups. Data are represented as mean ± SEM. * P < 0.05. (TIF) [file pntd.0008660.s005.tif]

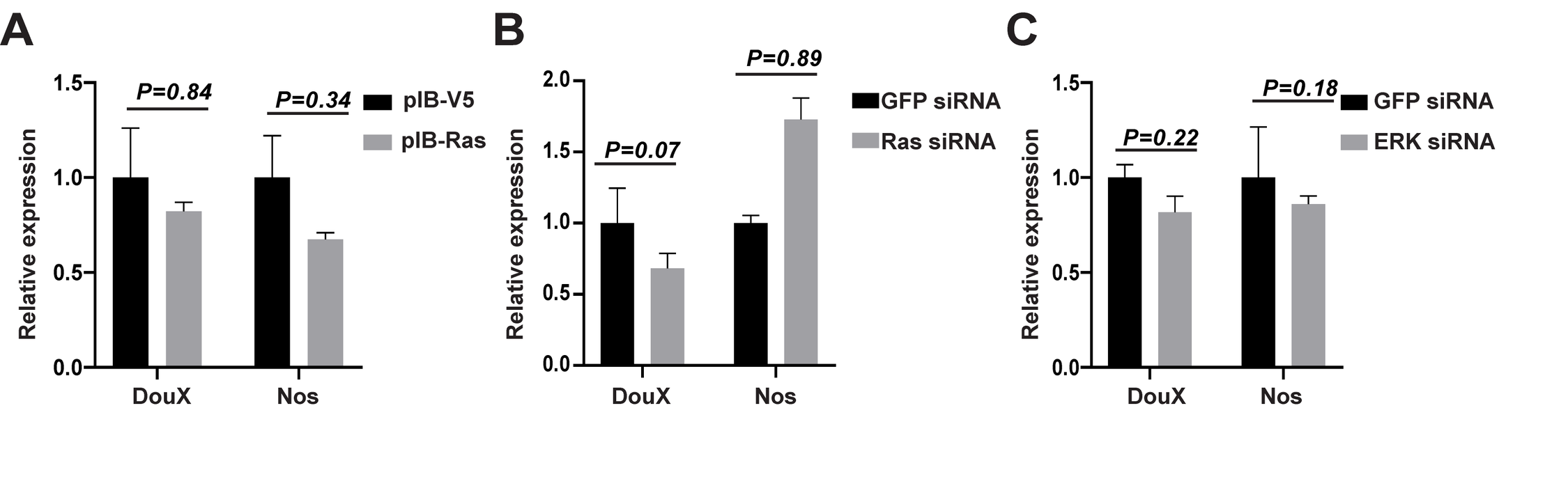

Supplement: S6 Fig — After transfection of pIB-Ras into Ae. albopictus C6/36 cells for 48 h, the mRNA level of Doux and Nos was detected by qPCR. pIB-V5 transfected cells were taken as the control. B, Ras knockdown shows little effect on the mRNA transcription of Doux and Nos. After transfection of Ras siRNA oligos individually into Ae. albopictus C6/36 cells for 48 h, the mRNA level of Doux and Nos was detected by qPCR. GFP siRNA was taken as the control. C, ERK knockdown shows little effect on the mRNA transcription of Doux and Nos. After transfection of ERK siRNA oligos individually into Ae. albopictus C6/36 cells for 48 h, respectively, the mRNA level of Doux and Nos were detected by qPCR. GFP siRNA was taken as the control. The experiments were repeated at least three times. Data are represented as mean ± SEM, t-tests. (TIF) [file pntd.0008660.s006.tif]

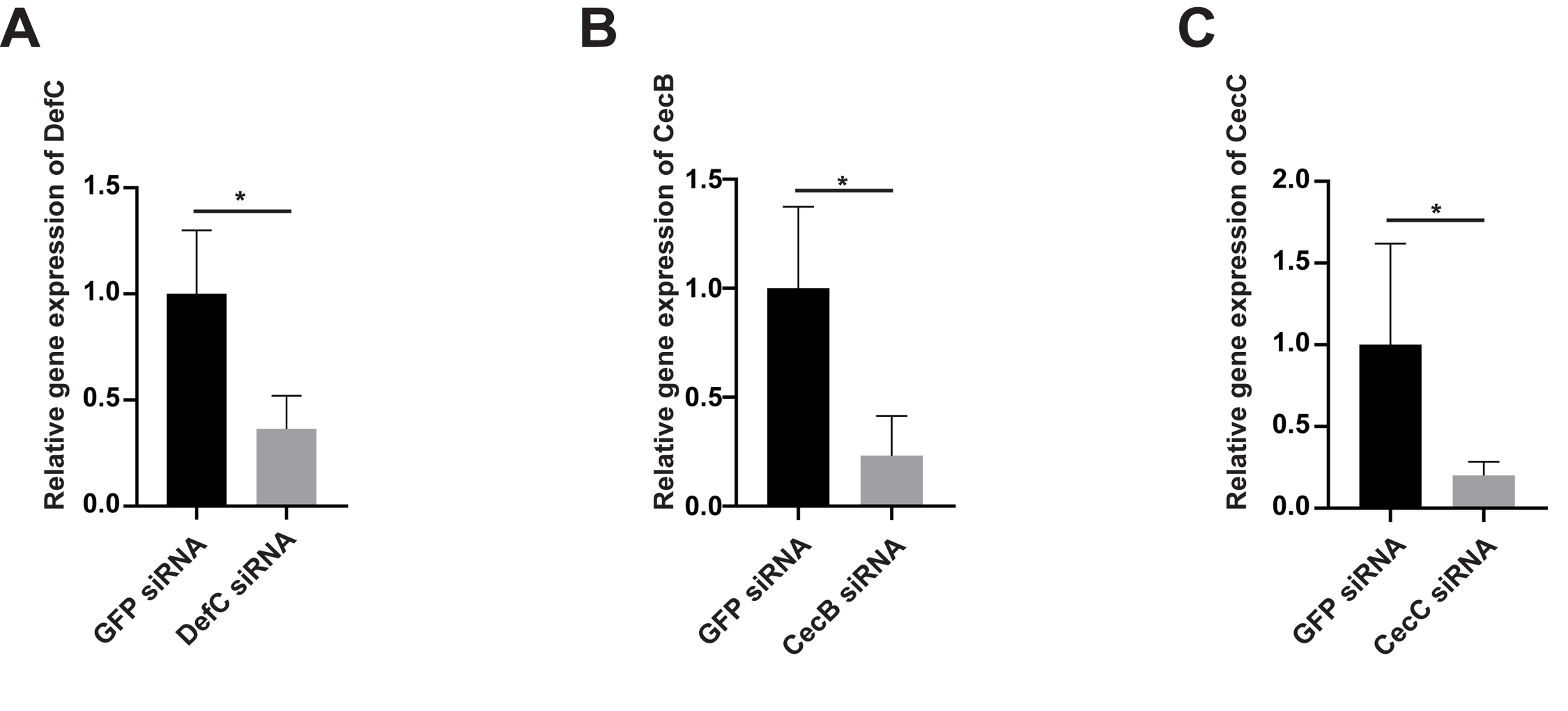

Supplement: S7 Fig — A-C, The siRNA oligos against Def C (A), Cec B (B) and Cec C (C) were transfected into Ae. albopictus C6/36 cells, respectively. After 48 h transfection, the mRNA level of Cec B, Cec C, and Def C were detected by qPCR. GFP siRNA was taken as the control. The experiments were repeated at least three times. Student’s t-tests were used to determine the significance of the difference between experimental and control groups in cell experiments. Data are represented as mean ± SEM. * P < 0.05. (TIF) [file pntd.0008660.s007.tif]

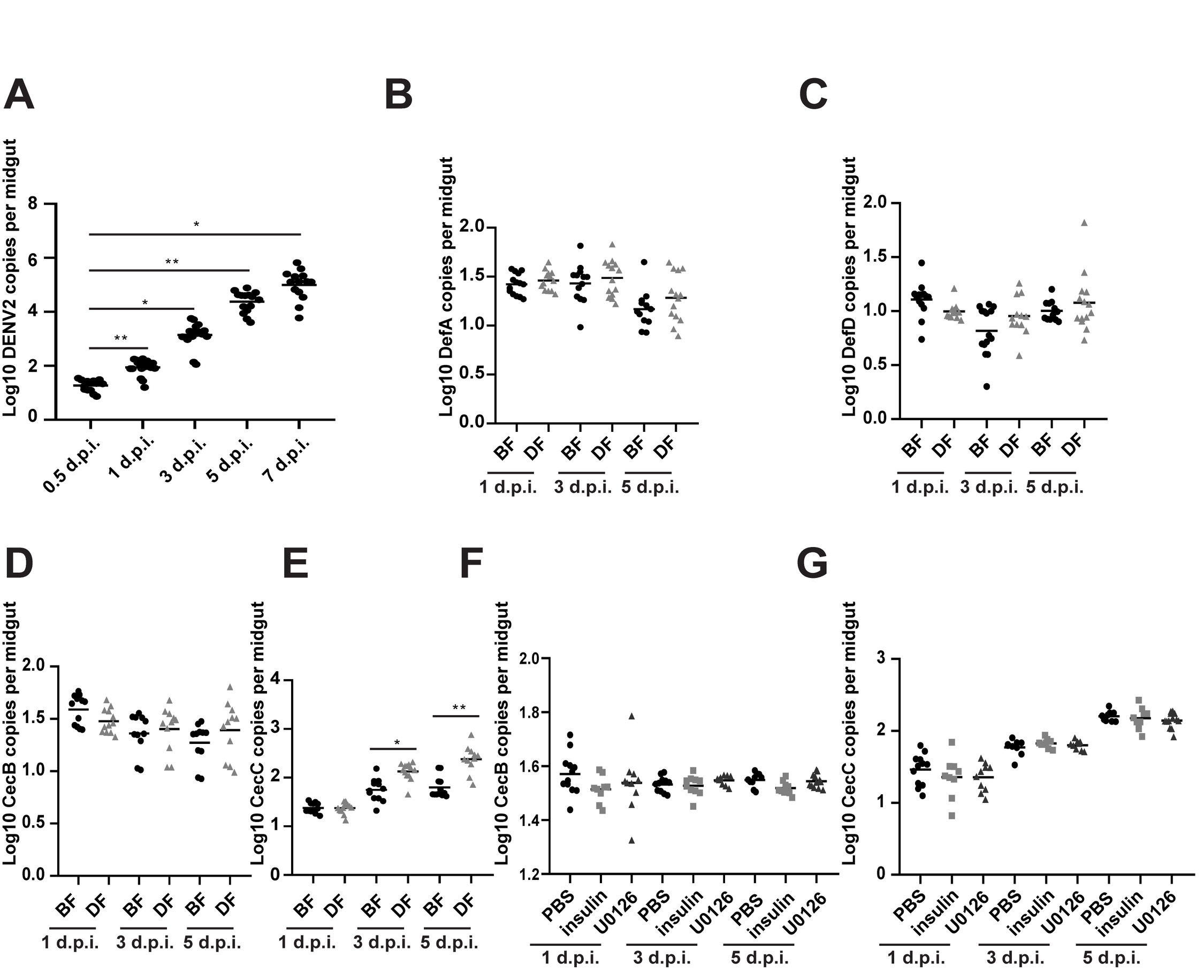

Supplement: S8 Fig — A, The transcription of DENV mRNA in the midguts of Ae. albopictus mosquitoes post-DENV2 infection. Feeding the female adult mosquitoes with the artificial blood containing DENV2, and the midgut samples from blood-fed mosquitoes were collected at 12 hours,1, 3, 5 and 7days post-infection. The viral mRNA levels in the midgut of blood-feeding mosquitoes were analyzed by qPCR. B-E, The expression of AMP genes in the midguts of Ae. albopictus mosquito post-DENV2 infection. Feeding the female adult mosquitoes with the artificial blood containing DENV2, and the midgut samples from blood-fed mosquitoes were collected at 1, 3 and 5 days post-infection. The AMP mRNA levels in the midgut of blood-feeding mosquitoes were analyzed by qPCR (B, Def A; C, Def D; D, Cec B; E, Cec C). F and G, insulin and U0126 treatments show no effect on the expression of Cec B and Cec C in the mosquito midguts. After feeding female mosquitoes with viral blood meals supplemented insulin and U0126, the midguts of blood-fed mosquitoes at 1, 3 and 5 days were dissected and used for qPCR analyses (F, Cec B; G, CecC). The viral blood meals supplemented PBS were taken as the control. DENV 2 RNA level and AMP genes expression in the midguts were determined using an absolute quantification qPCR method and presented in log10. Dot plots represent the viral genome (A) and AMP gene (B-G) copies for midgut samples. Black bars in the dot plots represent treatment medians. Each dot represents a single midgut sample. N = 16–20 samples per treatment. Mann-Whitney U test was used to determine P values for all comparisons. * P < 0.05, ** P < 0.01. (TIF) [file pntd.0008660.s008.tif]
