# Supplementary material for: The Ras/ERK signaling pathway couples antimicrobial peptides to mediate resistance to dengue virus in Aedes mosquitoes
Source: PLoS Negl Trop Dis. 2020 Aug 31;14(8):e0008660. doi: 10.1371/journal.pntd.0008660 (PMC7485967; doi:10.1371/journal.pntd.0008660)
Supplement: S3 Fig — The sequences of Sos, Ras, RAF, MEK, and ERK were aligned using Clustal X, and an unrooted phylogenetic tree was built with MEGA 7 software by using the neighbor-joining method. The bootstrap values of 1000 replicates (%) are indicated on the branch nodes. Ae. albopictus (Aal), Ae. aegypti (Aae), An. gambiae (Ag), Cx. quinquefasciatus (Cq) and Dm. melanogaster (Dm) are indicated, individually. (PDF) [file pntd.0008660.s003.pdf]

Phylogenetic analyses of the Ras/ERK signaling pathway in mosquitoes and *Drosophila*

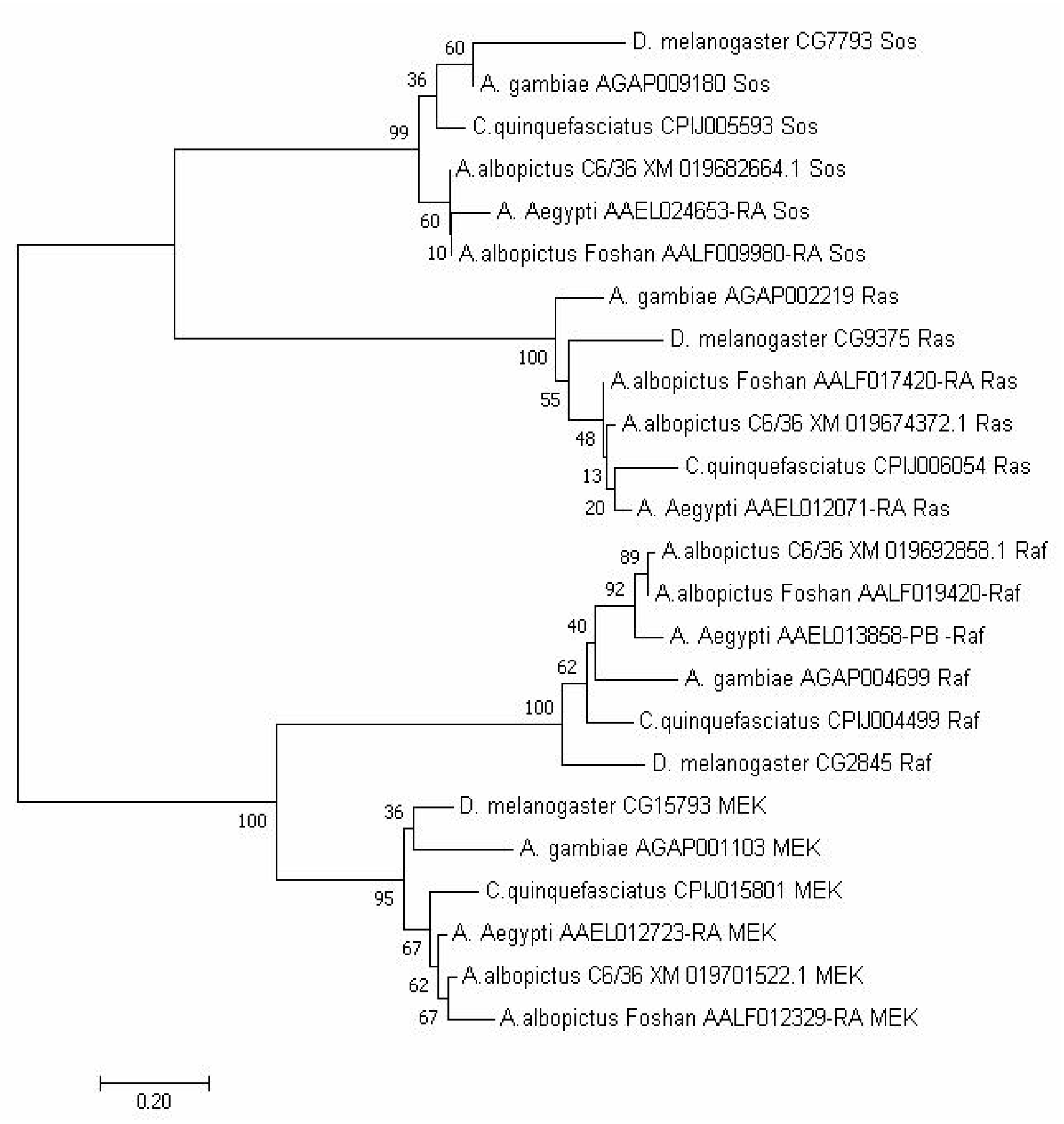

Multiple sequence alignment of Sos

```
Aae_Sos         MFAG------------------------------------------GQHVSITDATDYDF
Aal_Sos         MFAG------------------------------------------GQHVSITDATDYDF
Cq_Sos          MFAG------------------------------------------GQHVSITDATDYDF
Ag_Sos          MFSG------------------------------------------SSHVSITDAADYDF
Dm_Sos          MFSGPSGHAHTISYGGGIGLGTGGGGGSGGSGSGSQGGGGGIGIGGGGVAGLQDCDGYDF
                **:*                                          .  ..: *. .***

Aae_Sos         EKAENAAKWKGVFITSLRKVLEQVHPSLQAREDALLYVESLCLRLLAMLCAKPPPHTVLD
Aal_Sos         EKAENAAKWKGVFITSLRKVLEQVHPSLQAREDALLYVESLCLRLLAMLCAKPPPHTVVD
Cq_Sos          EKAENAAKWKGVFVTSLRKVLEQLHPSLQAREDALLYVESLCLRLLAMLCAKPPPHTVQD
Ag_Sos          ENAENAAKWRGLFISSLRKVLEQVHPSLQAREDALLYVESLCLRLLATLCAKPPPHTVMD
Dm_Sos          TKCENAARWRGLFTPSLKKVLEQVHPRVTAKEDALLYVEKLCLRLLAMLCAKPLPHSVQD
                 :.****:*:*:* .**:*****:** : *:********.******* ***** **:* *

Aae_Sos         VEERIGRTFPTPIDKWALSEARETIDKSKKKKSVLPVDRVHTLLQKEVLQYKIDSSVSLF
Aal_Sos         VEERIGRTFPTPIDKWALNEARETIDKSKKKKPVLPVDRVHTLLQKEVLQYKIDSSVSLF
Cq_Sos          VEDRIGRTFPTPIDKWALGEARDIIDKSKKKKPVLPVDRVHTLLQKEVLQYKIDSSVSLF
Ag_Sos          VEDRIIRTFPTPIDRWALGEARETIDKSKKKKPVLPVDRVHTLLQKEVLQYKIDSSVSLF
Dm_Sos          VEEKVNKSFPAPIDQWALNEAKEVIN-SKKRKSVLPTEKVHTLLQKDVLQYKIDSSVSAF
                **::: ::**:***:***.**:: *: ***:*.***.::*******:*********** *

Aae_Sos         LVAVLEYISADILKLAGNYVKNIRHIEISREDIEVAMCADK-------------------
Aal_Sos         LVAVLEYISADILKLAGNYVKNIRHIEISREDIEVAMCADK-------------------
Cq_Sos          LVAVLEYISADILKLAGNYVKNIRHIEISREDIEVAMCADKYLAKRIHHASHERLNRIVF
Ag_Sos          LVAVLEYISADILKLAGYYVKNIRHIEITREDIEVAMCADK-------------------
Dm_Sos          LVAVLEYISADILKMAGDYVIKIAHCEITKEDIEVVMNADR-------------------
                **************:** ** :* * **::*****.* **:

Aae_Sos         -----VLMDMFYQGDNSSSMAPSPLP-PTPRTSLSYEEVVKELIHDEKQYQRDLHMIIRV
Aal_Sos         -----VLMDMFYQGDNSSSMAPSPLP-PTPRTSLSYEEVVKELIHDEKQYQRDLHMIIRV
Cq_Sos          IPRTQVLMDMFYQGDSSSSMAPSPLP-PTPRTSLSYEEVVKELIHDEKQYQRDLHMIIRV
Ag_Sos          -----VLMDMFYQGESSNSMAPSPLP-PTPRASLSYEEVVKELIHDEKQYQRDLHMIIRV
Dm_Sos          -----VLMDMLNQ--SEAHILPSPLSLPAQRASATYEETVKELIHDEKQYQRDLHMIIRV
                     *****: *  ..  : ****. *: *:* :***.*********************

Aae_Sos         FREELVKIVKDPKELDPIFSNIMDIYEVSVTLLGSLEDVIEMSQEQTPPCIGSCFEELAE
Aal_Sos         FREELVKIVKEPKELDPIFSNIVDIYDLSVTLLGSLEDVIEMSQEQTPPCIGSCFEELAE
Cq_Sos          FREELVKIVRDPKELDSIFSNIMDIYEVSVTLLGSLEDVIEMSQEQTPPCIGSCFEELAE
Ag_Sos          FREELVKIVKDPKELDLIFSNIIDIYEVSVTLLGSLEDVIEMSQEQTPPCIGSCFEELAE
Dm_Sos          FREELVKIVSDPRELEPIFSNIMDIYEVTVTLLGSLEDVIEMSQEQSAPCVGSCFEELAE
                ********* :*:**: *****:***:::*****************:.**:*********

Aae_Sos         AAEFDVYAKYAKDITSVQAKEALANLLSRPEVLDSNSFRIHNRHPNPCDLFQANSLMSAG
Aal_Sos         AAEFDAYARYAKDITSVQAKEALANLLSRP---------------------EANSLMSAG
Cq_Sos          AAEFDVYAKYAKDITSVQAKEALANLLARP---------------------EANSLMSAG
Ag_Sos          AAEFDVYAKYAKDITSVTAKEALANLLARP---------------------EASSLMSAG
Dm_Sos          AEEFDVYKKYAYDVTSQASRDALNNLLSKP---------------------GASSLTTAG
                * ***.* :** *:**  :::** ***::*                      *.** :**
```

```
Aae_Sos           HGFREAVKFYLPKLLLGPIGHAQLYLDYIKILLQLSPSQEDKESFEQVQGLLKPLQCELQ
Aal_Sos           HGFREAVKFYLPKLLLGPIGHAQLYLDYIKILLQLSPSQEDKESFEQVQGLLKPLQCELQ
Cq_Sos            HGFREAVN----------------------------PLKEDKESFEQVQGLLKPLQCELQ
Ag_Sos            HGFKEAVKFYLPKLLLGPIGHAQLYLDYIKVLLQLSPSQEDKESFEQVQGLLKPLQCELQ
Dm_Sos            HGFRDAVKYYLPKLLLVPICHAFVYFDYIKHLKDLSSSQDDIESFEQVQGLLHPLHCDLE
                  ***::**:                            . ::* **********:**:*:*:

Aae_Sos           GISSLLPKEYFARVNSRARRQSAIEKTRDLQNSVEHW-DKDVGQCCNEFIREDTLAKLSS
Aal_Sos           GISSLLPKEYFARVNSRARRQSAIEKTRDLQNSVEHW-DKDVGQCCNEFIREDTLAKLSS
Cq_Sos            SISSLLPKEYFARVNSRARRQSAIEKTRDLQNSVEHW-DKDVGQCCNEFIREDTLAKLSS
Ag_Sos            SISSLLPKEYFTRVNSRARRQSAIEKTRDLQNTVEHW-DKDVGQCCNEFIREDTLAKLSS
Dm_Sos            KVMASLSKERQVPVSGRVRRQLAIERTRELQMKVEHWEDKDVGQNCNEFIREDSLSKLGS
                   : : *.**  . *..*.*** ***:**:** .**** ****** ********:*:**.*

Aae_Sos           GRRQ-TERKVFLFDGLLVLCKT--RRQIVPG--TNYDYRLKERFFMRKVEIIDRPDTEEL
Aal_Sos           GKRQ-TERKVFLFDGLLVLCKT--RRQIVPG--NNYDYRLKERFFMRKVEIIDRPDTEEL
Cq_Sos            GKRQ-TERKVFLFDGLLVLCKT--RRQIVPG--NNYDYRLKERFFMRKVEIIDRSDTDEL
Ag_Sos            GKRQ-TERKVFLFDGLLVLCKA--RRQIVPG--NNYDYRQKERFFMRKVEIIDRPDTDEL
Dm_Sos            GKRIWSERKVFLFDGLMVLCKANTKKQTPSAGATAYDYRLKEKYFMRRVDINDRPDSDDL
                  *:*  :**********:****:  ::*  ..  . **** **::***:*:* **.*:::*

Aae_Sos           KYAFEISPREPTSVVLIAKNAQHKNDWMADLVMLNTKSMLERILDSILLDIEKKHPLKVP
Aal_Sos           KYAFEISPREPTSVVLIAKNAQHKNDWMADLVMLNTKSMLERILDSILLDIEKKHPLKLP
Cq_Sos            KYAFEISPREPTSVVLIAKNAQHKNDWMADLVMLNTKSMLERILDSILLDIEKKHPLKLP
Ag_Sos            KHAFEISPREQQSVVLITKNAQHKNDWMADLIMLNTKSMLERILDSILLDIEKKHPLRLP
Dm_Sos            KNSFELAPRMQPPIVLTAKNAQHKHDWMADLLMVITKSMLDRHLDSILQDIERKHPLRMP
                  * :**::**   .:** :******:******:*: *****:* ***** ***:****::*

Aae_Sos           SPDIYAFAVPDSPSNIVLEEREGTGVPLIKGATLTKLIERLTYHIYADPMFVRTFLTTYR
Aal_Sos           SPDKYAFAVPDSPSNIVLEEREGTGVPLIKGATLTKLIERLTYHIYADPMFVRIFLTTYR
Cq_Sos            TPEIYAFAVPDSPSNIVLEEREGTGVPLIKGATLTKLIERLTYHIYADPMFVRTFLTTYR
Ag_Sos            SPDMYKFAVPDGPSNIVLEEREGTGVPLIKGATLCKLIERLTYHIYADPMFVRTFLTTYR
Dm_Sos            SPEIYKFAVPDSGDNIVLEERESAGVPMIKGATLCKLIERLTYHIYADPTFVRTFLTTYR
                  :*: * *****. .********.:***:****** ************** *** ******

Aae_Sos           SFCSPTELLRLLVERFDIPDPALVYDSASDKDLG----PDTDKFHKNSQREDWKRYKKEY
Aal_Sos           SFCSPKELLRLLVERFQIPEPGQVNDSASDKDLV----SDTDKFHKNSQREDVKRFKKEY
Cq_Sos            SFCSPRELLRLLVERFDIPDPTLVYDSAADKDIG----SDLDKFHKNSQREDWKRYKKEY
Ag_Sos            SFCSPKELLQLLVERFDIPDPAVVYDPAAEKELA----GDTDKFHKNSQREDWKRYKKEY
Dm_Sos            YFCSPQQLLQLLVERFNIPDPSLVYQDTGTAGAGGMGGVGGDKEHKNSHREDWKRYRKEY
                   **** :**:******:**:*  * : :.          . ** ****:*** **::***

Aae_Sos           VQPVQFRVLNVLRHWVDHHFYDFERDSELLEELLKFLETVRGKSMRKWVDSVLKIVQRKT
Aal_Sos           MQPVQFRVLNVLRHWVDHHFYDFERDSELLEELLKFLETVAGKPMRKWVDSVLKIVQRKN
Cq_Sos            VQPVQFRVLNVLRHWVDHHFYDFERDSELLDELLRFLETVRGKSMRKWVDSVLKIVQRKN
Ag_Sos            VQPVQFRVLNVLRHWVDHHFYDFERDGELLESLERFLETVRGKSMRKWVDSVMKIVQRKN
Dm_Sos            VQPVQFRVLNVLRHWVDHHFYDFEKDPMLLEKLLNFLEHVNGKSMRKWVDSVLKIVQRKN
                  :***********************:*  **:.* .*** * **.********:******.

Aae_Sos           ESEDSHRQITFAFGHSPPAIEHHLPLTIGEEEYNLLMLHPLELARQLTLLEFEMYKNVKP
Aal_Sos           ESEESHRQITFAYGNSPPAIEHHLPLNIGENEYNLLILHPLELARQLTLLEFEMYKNVKP
```

```
Cq_Sos          ESEDNHRQITFAFGHSPPAIEHHLPLSS-EHEFNLLTLH---------------------
Ag_Sos          ESEDNHRQITFAFGHSPPAIEHHLPLNG-ENEFNLLMLHPLELARQLTLLEFEMYKNVKP
Dm_Sos          EQEKSNKKIVYAYGHDPPPIEHHLSVPN--DEITLLTLHPLELARQLTLLEFEMYKNVKP
                *.*..:::*.:*:*:.**.*****.:    .* .** **

Aae_Sos         SELVGSVWTGKDKETTSPNLLRIMHHTTNFTRWIEKSIIEAENFDERVAMASRAIEVMMV
Aal_Sos         SELVGSVWTGKDKETTSPNLLRIMHHTTNFTRWIEKSIIEAENFDERVAMASRAIEVMMV
Cq_Sos          ----RSSWP------------------AIYPDWIEKSILEAENFEERVAMASRAIEVMMV
Ag_Sos          SELVGSVWTGKDKETTSPNLLKIMHHTTNFTRWIEKSIIEAENFEERVAMASRAIEVMMV
Dm_Sos          SELVGSPWTKKDKEVKSPNLLKIMKHTTNVTRWIEKSITEAENYEERLAIMQRAIEVMMV
                     * *.                  :  . ****** ****::**:*: .********

Aae_Sos         LQDLNNFNGVLSIVSAFQGAAVHRLKLTLEEIPKSYQRVLAECRELNNSHFKKYQEKLRS
Aal_Sos         LQDLNNFNGVLSIVSAFQGAAVHRLKLTLEEIPKSYQRALAECRELNDSHFKKYQEKLRS
Cq_Sos          LQDLNNFNGVLSIVSAFQGAAVHRLKLTLEEISKSYQKVLAECGELNNSHYRKYQEKLQ-
Ag_Sos          LQDLNNFNGVLSIVSAFQGAAVHRLKLTLEDIPKRHQQVLAECRELNNSHFKKYQEKLRS
Dm_Sos          MLELNNFNGILSIVAAMGTASVYRLRWTFQGLPERYRKFLEECRELSDDHLKKYQERLRS
                : :******:****:*:  *:*:**: *:: :.: ::: * ** **.:.* :****:*:

Aae_Sos         INPPCVPFFGMYLTNILHIEEGNPDFLPKTKLINFSKRRRVAEITGEIQQYQNQPYCLKV
Aal_Sos         INPPCVPFFGMYLTNILHIEEGNPDFLPNTELINFSKRRRVAEITGEIQQYQNQPYCLKV
Cq_Sos          ------------------------------------------------------------
Ag_Sos          INPPCVPFFGMYLTNILHIEEGNPDFLPNTELINFSKRRRVAEITGEIQQYQNQPYCLKV
Dm_Sos          INPPCVPFFGRYLTNILHLEEGNPDLLANTELINFSKRRKVAEIIGEIQQYQNQPYCLNE


Aae_Sos         DPSIRHFLENLDPFTGMSVTEIQNYLYEESKRIEPKNCRQPLKFARKWPDISLKSPGIKP
Aal_Sos         DKKIRPFLENLDPFKGMSVTEIQNYLYEESKRIEPKNCRQPLKFARKWPDISLKSPGIKP
Cq_Sos          ---------NLDPFKGMSVTEISNYVYEESKRIEPKNCRQPLKFPRKWPDIPLKSPGIKP
Ag_Sos          DPSIRNFLENLDPFKGMSITEIQNYLYEESKRIEPKNCRQPLKFPKKWPDISLKSPGIKP
Dm_Sos          ESTIRQFFEQLDPFNGLSDKQMSDYLYNESLRIEPRGCKTVPKFPRKWPHIPLKSPGIKP
                         :****.*:* .::.:*:*:** ****:.*:   **.:***.*.********

Aae_Sos         SSRRHNSSANSSLNLTGTLPFTNKAPLGNSN----DTGAEQQSPTASSSSSSVLDYSIFA
Aal_Sos         --RRHNSSANSSLNLTGTLPFINKAPLGNNNNIDTGSGAEQQSPTASSSSSSVLDYSIFA
Cq_Sos          SSRRNNSSANSSMTLTGTLPFSKTSLLNNND-----TGGEQ-SPPANSSSSSVHDYSIFA
Ag_Sos          SSRRNNSSANSSMTLPGTLPYSKTSLLANSD------TGEQ-SPPASSSSS-VHDYSIFA
Dm_Sos          --RRQNQ-TNSSSKLSNSTSSVAAAAAASST-----ATSIATASAPSLHASSIMDAPTAA
                  **:*. :*** .*..: .     :   ..          .    :....   :* : * .  *

Aae_Sos         PVNIHSSSSSQ-------HHQYHHHQQQMYNYQHHSQGSMQYYPGHHQSGGGQQQNHHHY
Aal_Sos         PVNITNPSWTTSRHVFPYHQDPDKHMPPLHVIYPHRHNQLPPVPSSSLAAGGQQQNNHHF
Cq_Sos          PVNIHSSSSTSTSS--SQYYQHHHQQQQHHSFYQQHHSITQGYNSCRKATSSSATS----
Ag_Sos          SVNIHSSSSGTIVS-------MSHNDATVQQQQQQGSAVVIPHPDIPPAISPRTDKPQ--
Dm_Sos          AANAGSGTLAGEQS-----PQHNPHAFSVFAPVIIPERNTSSWSGTPQHTRTDQNNGEVS
                ..*  . :                :                   .          .

Aae_Sos         N--HHGHQHNLSQSVVNLASTADHQMAPEIPRRSDSIILTP-SLSSQVQYNLNNSSLSES
Aal_Sos         YNYHHGHQHSLSQSVVNLASTADQQMAPEIPRRSDSIILTTSSLSSHTQYNLNNSSLSES
Cq_Sos          ---PPSQGYSFSQSVVNLASASDQQMAPEIPRRSDSIILTH------APYNLNNSSLSES
Ag_Sos          ---PLHPPPPPPPSLQHAASASPGATAGSGSSIAGSSILAN--MAARTTVVLAFARLSFQ
```

```
Dm_Sos              ---VPAPHLPKKPGAHVWANNNSTLASASAMDVVFSPALPEHLPPQSLPDSNPFASDTEA
                                 .    *.      : .      *  *.              :  :

Aae_Sos             SSNTG-----GGSKLVPSPRYPSSSMSALSSSSSGISVSSFYGIMSASNSDGNSNNNPTT
Aal_Sos             SSNTGDGGGVGGVRLHPSPRYPSSSMSALSSSSSGISSSSIYGVMSASNSD---------
Cq_Sos              SGNPCGSGG-GSGKLVPSPRNPYSSMSALSSSSSGISVSSYFGILSASSSDGNSNNNHST
Ag_Sos              PENPAP----------ASSHRGSGSATCLPAPPSPCRPS---------------------
Dm_Sos              PPSPLP-------KLVVSPRHETGNRSPFHGRMQNSPTHS--------------------
                    . ..             *.:   .. : : .  .

Aae_Sos             AS-FKHTPLLSQNKVVAN--TCAEDSPHSILSASGVGGGGSLS--MSSSSATNAASIVSL
Aal_Sos             --------------------------------ASGVGGGGSLS--LSSSSSTNAASIVSL
Cq_Sos              ATSFKLTPMLSQPPKVVSNTQHAEDTPNSILSASGLGGGGGGGGMASSSAATNATSIVSL
Ag_Sos              --------------------------------SSHRCGGCGRWWWAGGQFDPVQQQRVTF
Dm_Sos              --------------------------------TASTVTLTGMSTSGGEEFCAGGFYFNSA
                                                    ::      .     . .  .      :

Aae_Sos             SEGSAVPQP-----------NTGTAISPCTTMSSPSASSSS-TVTAVGT---------TV
Aal_Sos             SDGPAVSQPPPP-------NAAGMATSPCTTISSPSASSSSTAVTAVGVGP----SYGAG
Cq_Sos              SDGPTAPLPPLSSVPPSNNNPTGSGNSSSVSSSASASSSSTVVVSSTAYGAGTQSNLPPP
Ag_Sos              RQQPPSVSP-----------VCGRCGRRRQLVEQCRHWSDT-----------------DL
Dm_Sos              HQGQPGAVP-----------ISPHVNVPMATNMEYRAVPPPLPPRRK-----------ER
                     :  .   *                             . .

Aae_Sos             TASPAPASSGAADIPPAISPRTDKPVYPPGPPPLRNHGGG--ATEFCYNTS------GLP
Aal_Sos             ATTVFAASPSSTDIPPAISPRTDRPVCPP-PAPSRNHGGGSGPTEFSYNTS------GLP
Cq_Sos              PPTPSSAASGVGDIPPAISPRTDKPAHPPAPPPLRNNGGG-GGTEFCYNMSSSSSSFGLP
Ag_Sos              TARQRTKYAQCATTPGASTTSGAKRIFHGFDPVVADAAGAGRTDATTERRS---------
Dm_Sos              TESCADMAQKRQAPDAPTLPPRDGELSPPPIPPRLNHSTGISYLRQSHGKS---------
                    .               .  .           .   : . .          *

Aae_Sos             --TSPLATS----CNFRISSSNIGKNQNCDFTTASSAGNRSSCDNSPIFPSSPQMHPIHG
Aal_Sos             --TSPLATAG---SSFRISSSNTVRNQNCDFTTAT--GNHSSCENSPIFPSSPHILPMGG
Cq_Sos              NCTSPHATSSSSSSSFRNNSTNPLKNQNCDFTSASAALNRSSCENSPIYPSSPKMYFS--
Ag_Sos              -----AATT----ADLHTVVTLPVRSVQPGRHDAATLHAKSECVR--------RWWGIDV
Dm_Sos              -------------KEFVGNSSLLLPNTSSIMIRRNSAIEKR-------------------
                                  .:    :    . .           :

Aae_Sos             GTASTMDFNNCAISPTLSTSVSSTSSVYQTTYN-NSSTTADSMILNTTAASN--------
Aal_Sos             GQASVMDFNNCATSPTLSTSVSSTSSVYQTTFN-ASSTTADSMILNTTVASN--------
Cq_Sos              -TTAAAGTSSGMTDFNYSSSASAVSPSLSTSYNSASSTTADSMILNATTSGTGMSGGGGY
Ag_Sos              FVLVLVDANAGQFEPAQSAQSAAANAAPYQHDHDAAQLGDGSGGEGKHSQHC--------
Dm_Sos              --AAATSQPNQAAAGPISTTLVTVSQAVATDEPLPLPISPAASSSTTTSPLT--------
                          .          *:   :..                :

Aae_Sos             ------DLGP-PHSPHVNVPNTHNIPPPMPPPLPPRVKRKESSGESSQSSQVR-QAPDAP
Aal_Sos             ------DLGP-PHSPHVNVPNTHNIPPPMPPPLPPRVRRRESSGESSQSAQVR-QAPDAP
Cq_Sos              GGSSSGDFGPIPISPHVNVPNAHNIPPPMPPPLPPRVKRKESSGESSQSSQVR-QAPDAP
Ag_Sos              ------HFELAVRAVDDRWRQRRWWWCRWSERGPGRWGRWQWIDNELRRSQQQ-VGAELT
Dm_Sos              -------PAMSPMSPNIPSHPVESTSSSYAHQLRMRQQQQQQTHPAIYSQHHQHHATHLP
                                 : .      .      .     *  : :         : :  ... .
```

```
Aae_Sos         KLPPRDISPPPVPPRIHNPSHQICGNVQQQHQLLHSVGAC-DSCNLVNVLPKDESPSSSS
Aal_Sos         RLPPRDISPPPLPPRIHSHSHYSFGNVQQQQQLAHSSGAF-DSWNHINVLP-DESPSSSS
Cq_Sos          RLPPRDLSPPPLPPRTHIQSHYLFGNVQQQQQLYNSSCAVSDSWNHGHQFPVS-------
Ag_Sos          HLGAGAAAECTLPKGQYKHFAPLFARRNDTKTTPQTETEQYKFRYVSYNVSLSQHEL---
Dm_Sos          HHPHQHHSNPTQSRSSPKEFFPIATSLEGTPKLPPKPSLSANFYNNPDKGTMFLYPSTNE
                :      :  . .              :       .     .        .

Aae_Sos         SSSTLTRDNLNQHNMSE-QRLLMLPHTSTIMMRRNSAMDRASKEKVSNITASPSLSGLSS
Aal_Sos         SSSTLTRDNLNQHNMSTTQQALMLPHTSTIMMRRNSAMDRGSREKVPNIAASPSLSGLSS
Cq_Sos          ------------------------------------------------------------
Ag_Sos          ------------------------------------------------------------
Dm_Sos          E-----------------------------------------------------------


Aae_Sos         TVGGVVG----GGSTASSGPVSLSSSS--APPPPCGAKNKSVQNSPVSQQQQQQPAVCGR
Aal_Sos         ATATTTAVGGAAGTSVGSGPVSLPSTSGVAPPPPCGTKNKSVQNSPVSQQQQ--PPVRDR
Cq_Sos          ------------------------------------------------------------
Ag_Sos          ------------------------------------------------------------
Dm_Sos          ------------------------------------------------------------


Aae_Sos         RPSTNISPRFSPGETTPKLPPKPKQTNLSSHSDRTMFPYPSTN
Aal_Sos         RTSTNTSPRFSPGETTPRLPPKPKQTNQSSHPDRTMFQYPSTN
Cq_Sos          -------------------------------------------
Ag_Sos          -------------------------------------------
Dm_Sos          -------------------------------------------
```

Multiple sequence alignment of Ras

```
Cq_Ras          MTEYKLVVVGAGGVGKSALTIQLIQNHFVDEYDPTIEDSYRKQVVIDGETCLLDILDTAG
Ag_Ras          MTEYKLVVVGAGGVGKSALTIQLIQNHFVDEYDPTIEDSYRKQVVIDGETCLLDILDTAG
Aal_Ras         MTEYKLVVVGAGGVGKSALTIQLIQNHFVDEYDPTIEDSYRKQVVIDGETCLLDILDTAG
Aae_Ras         MTEYKLVVVGAGGVGKSALTIQLIQNHFVDEYDPTIEDSYRKQVVIDGETCLLDILDTAG
Dm_Ras          MTEYKLVVVGAGGVGKSALTIQLIQNHFVDEYDPTIEDSYRKQVVIDGETCLLDILDTAG
                ************************************************************

Cq_Ras          QEEYSAMRDQYMRTGEGFLLVFAVNSAKSFEDIGTYREQIKRVKDAEEVPMVLVGNKCDL
Ag_Ras          QEEYSAMRDQYMRTGEGFLLVFAVNSAKSFEDIGTYREQIKRVKDAEEVPMVLVGNKCDL
Aal_Ras         QEEYSAMRDQYMRTGEGFLLVFAVNSAKSFEDIGTYREQIKRVKDAEEVPMVLVGNKCDL
Aae_Ras         QEEYSAMRDQYMRTGEGFLLVFAVNSAKSFEDIGTYREQIKRVKDAEEVPMVLVGNKCDL
Dm_Ras          QEEYSAMRDQYMRTGEGFLLVFAVNSAKSFEDIGTYREQIKRVKDAEEVPMVLVGNKCDL
                ************************************************************

Cq_Ras          QAWAVDMNQARDVAKQYGVPFVETSAKTRMGVDDAFYTLVREIRKDKE-RGKKNKKHNKL
Ag_Ras          QAWAVDMNQARDVAKQYGVPFVETSAKTRMGVDDAFYTLVREIRKDKE-RGKKNRKHHKL
Aal_Ras         QAWAVDMNQARDVAKQYGVPFVETSAKTRMGVDDAFYTLVREIRKDKE-RGKKNRKHNKL
Aae_Ras         QAWAVDMNQARDVAKQYGVPFVETSAKTRMGVDDAFYTLVREIRKDKE-RGKKNRKHNKL
Dm_Ras          ASWNVNNEQAREVAKQYGIPYIETSAKTRMGVDDAFYTLVREIRKDKDNKGRRGRKMNKP
                 :* *: :***:******:*::*************************: :*::.:* :*
```

```
Cq_Ras          GSSRRFKCRLL
Ag_Ras          VSSRRFKCQLL
Aal_Ras         GSSRRFKCRLL
Aae_Ras         GSSRRFKCRLL
Dm_Ras          --NRRFKCKML
                  .*****::*
```

Multiple sequence alignment of Raf

```
Aae_Raf         MAVFDDSGSSSSSTNNNNNSSSNNIICKVKNSNGKIVVGSSTSSSNST--------STTI
Aal_Raf         MAVFDDSGSSSSSTNNNN---SNNIICKVKNSNGNSVVGSSTSSSNSTSKITSKGASTAA
Cq_Raf          ------------------------------------------------------------
Ag_Raf          ------------------------------------------------------------
Dm_Raf          ------------------------------------------------------------


Aae_Raf         TSKGGGTVPSLSLYED--GNLRIMSRTDTDDTDDEQQLEALDPVSQLEEDLRNIKSVIHV
Aal_Raf         AAKGTGKVPNLSLYEDNLRTARTMS-ADTDDTDDEQQLEALDPYSQLEEDLRNIKSVIHV
Cq_Raf          -----------------------MS-VETDDTDDEQQQLALDPLSQLEEDLRNIRSVILV
Ag_Raf          -----------------------MSSADAEDTDEE-----LDAFLQLEEDLRNIKSVIHV
Dm_Raf          -----------------------MS-SESSTEGDS------DLYDPLAEELHNVQLVKHV
                                       **  ::.  .:.      *    * *:*:*:: *  *

Aae_Raf         TRENIDALNAKFADFQQP-----------------------------------------P
Aal_Raf         TRENIDALNAKFADFQQP-----------------------------------------P
Cq_Raf          TRENIDALNAKFADFQQPPALYLEENAQMFEFGRNCIACGSIYMSFLQSYEKICFRFLPP
Ag_Raf          TRENIDALNEKFADFQQP-----------------------------------------P
Dm_Raf          TRENIDALNAKFANLQEP-----------------------------------------P
                ********* ***::*:*                                         *

Aae_Raf         ALYLEEYQELTSKLHDLETKEQELMERKSQMQAQ--ALAAQSERESTSEPSEPPDP-EER
Aal_Raf         ALYLEEYQELTSKLHELETKEQELMERKSQMQQARVQAAQQSERESTSEPSEPPDP-EER
Cq_Raf          ALYLEEYQELTSKLHDLETKEQDLGERRSQMRASQ-QQQQSAERESTSEPSEPPDPAEER
Ag_Raf          ALYLEEYQELTSKLHDLEIKEQDLMEKKIQMQNEH-------------------------
Dm_Raf          AMYLIEYQELTSKLHELEAKEQELMERLNSQDQQEDSSLVERFKEQPHYQNQTQILQQQR
                *:** **********:** ***:* *:  .

Aae_Raf         VEVDS-----------------MCGTLSRQSKMLLRAHLPNQQRTSVQVVPGMRLKDALAK
Aal_Raf         VEVDS-----------------MCGTLSRTSKMLLRAHLPNQQRTSVQVVPGMRLKDALAK
Cq_Raf          VEVSVTVGRGSVFVVGGF-GHMCGTLSRSSKMLLRAHLPNQQRTSVHLIPGMRLKDALAK
Ag_Raf          ----------------------MCGTLSRQSKMLLRAFLPNQQRTSVQVIPGMRLKDALAK
Dm_Raf          QLARVHHGNDLTDSLGSQPGSQCGTLTRQPKILLRAHLPNQQRTSVEVISGVRLCDALMK
                                      ****:* .*:****.*********.::.*:** *** *

Aae_Raf         ALKRRNLTCDICEVTSANS-DYPIHWDTDVSMLNCEEVFVRILDIG-FPTYISHQFIRKT
Aal_Raf         ALKRRNLTCDICEVTSANS-DYPIHWDTDVSMLNCEEVFVRILDIG-FPTYISHQFIRKT
Cq_Raf          ALKRRNLTADICEVTSSNS-DYPIPWDTDVCDLHCEEVIVRILDIG-FPTYISHQFIRKT
Ag_Raf          ALKRRNLTCEFCEVTAGNS-NYPIPWETDVSALNCDEVFVRILDIG-FPTYISHQFIRKT
Dm_Raf          ALKLRQLTPDMCEVSTTHSGRHIIPWHTDIGTLHVEEIFVRLLDKFPIRTHIKHQIIRKT
```

```
                  *** *:** ::***:: :*  : * *.**:  *: :*::**:**   : *:*.**:****

Aae_Raf           FFSLAFCECCRRLLFTGFYCNQCNYRFHQRCVDKVPLVCSKRHMDNTFYHHLLANPESTV
Aal_Raf           FFSLAFCECCRRLLFTGFYCNQCNYRFHQRCVDKVPLVCSKRHMDNTFYHHLLANPESTV
Cq_Raf            FFSLAFCECCRRLLFTGFYCNQCNYRFHQRCVDKVPLVCSKRHMDNTFYHHLLANPESTV
Ag_Raf            FFSLAFCECCRRLLFTGFYCNQCNYRFHQRCVDKVPPICSKRHMDSTFYHVLLANPESTA
Dm_Raf            FFSLVFCEGCRRLLFTGFYCSQCNFRFHQRCANRVPMLCQPFPMDSYYQLLLAENPDNGV
                  ****.*** ***********.***:******.::** :*.    **.  :    *  **:. .

Aae_Raf           GIINPG----------------TGGYNTSLRHPRS------------LNQQDRSNSAPN
Aal_Raf           GIINPG----------------TGGYNTSLRHPRS------------LNQQDRSNSAPN
Cq_Raf            GIINPG----------------TGGYSTSLRHPRS------------LNPQDRSNSAPN
Ag_Raf            GIINPG----------------AGGYSTSLRHPRS------------LNQHDRSNSAPN
Dm_Raf            GFPGRGTAVRFNMSSRSRSRRCSSSGSSSSSKPPSSSSGNHRQGRPPRISQDDRSNSAPN
                  *: . *                :.* .:* : * *             :. .********

Aae_Raf           VCINSV-MKPLFGAVDNRPLINCRPLQVAQANQEHSHSTQASPTNTLKHSKRPRARSADE
Aal_Raf           VCINSV-MKPLFGAVDNRPLINCRPLQVAQANQEHSHSTQASPTNTLKHSKRPRARSADE
Cq_Raf            VCINNVNMKPLFGLDAGRPLLNRGPLQVTRVLTEHSHSTQASPTNTLKHSKRPRARSADE
Ag_Raf            VCINNV-IKPFFGGE-NRHVISNRPLQAQ-PNQEHSHSTQASPTNTLNHSKRPRARSADE
Dm_Raf            VCINNI---RSVTSEVQRSLIMQARPPLPHPCTDHSNSTQASPTSTLKHN-RPRARSADE
                  ****.:      .      * ::             :**:*******.**:*. *********

Aae_Raf           SNKNLLSPRDSKQSEENWNIQAEEILIGQRIGSGSFGTVYKAHWHGPVAVKTLNVKTPSS
Aal_Raf           SNKNLLSPRDSKQSEENWNIQAEEILIGQRIGSGSFGTVYKAHWHGPVAVKTLNVKTPSS
Cq_Raf            SNKNLLSPRDPKQSEENWNIQAEEILIGQRIGSGSFGTVYKAHWHGPVAVKTLNVKTPSA
Ag_Raf            SNKNLLSPRDSKQSDENWNIQAEEILIGQRIGSGSFGTVYKAHWHGPVAVKTLNVKTPSP
Dm_Raf            SNKNLL-LRDAKSSEENWNILAEEILIGPRIGSGSFGTVYRAHWHGPVAVKTLNVKTPSP
                  ******  **.*.*:***** ******* ***********:*****************.

Aae_Raf           AQLQAFKNEVAMLKKTRHCNILLFMGCVSKPSLAIVTQWCEGSSLYKHIHVIETKFKLNT
Aal_Raf           AQLQAFKNEVAMLKKTRHCNILLFMGCVSKPSLAIVTQWCEGSSLYKHIHVIETKFKLNT
Cq_Raf            AQLQAFKNEVAMLKKTRHCNILLFMGCVSKPSLAIVTQWCEGSSLYKHIHVNETKFKLNT
Ag_Raf            AQLQAFKNEVAMLKKTRHCNILLFMGCVSKPSLAIVTQWCEGSSLYKHIHVNETKFKLNT
Dm_Raf            AQLQAFKNEVAMLKKTRHCNILLFMGCVSKPSLAIVTQWCEGSSLYKHVHVSETKFKLNT
                  ************************************************:** ********

Aae_Raf           LIDIARQAAQGMDYLHAKNIIHRDLKSNNIFLHDDLSVKIGDFGLATAKVRWSGSQQSNQ
Aal_Raf           LIDIARQAAQGMDYLHAKNIIHRDLKSNNIFLHDDLSVKIGDFGLATAKVRWSGSQQSNQ
Cq_Raf            LIDIARQAAQGMDYLHAKNIIHRDLKSNNIFLHDDFSVKIGDFGLATAKVRWSGSQQSNQ
Ag_Raf            LIDIARQAAQGMDYLHAKNIIHRDLKSNNIFLHDDFSVKIGDFGLATAKVRWSGSQQSNQ
Dm_Raf            LIDIGRQVAQGMDYLHAKNIIHRDLKSNNIFLHEDLSVKIGDFGLATAKTRWSGEKQANQ
                  ****.**.*************************:*:*************.****.:*:**

Aae_Raf           PTGSILWMAPEVIRMKEVNPYSFQSDVYAFGIVLYEMLTEQLPYSHINNKDQILFMVGCG
Aal_Raf           PTGSILWMAPEVIRMKEVNPYSFQSDVYAFGIVLYEMLTEQLPYNHINNKDQILFMVGCG
Cq_Raf            PTGSILWMAPEVIRMKEQNPYSFQSDVYAFGIVLYEMLTESLPYSHINNKDQILFMVGCG
Ag_Raf            PTGSILWMAPEVIRMKEQNPYSFQSDVYAFGIVLYEMLTEQLPYNHINNKDQILFMVGCG
Dm_Raf            PTGSILWMAPEVIRMQELNPYSFQSDVYAFGIVMYELLAECLPYGHISNKDQILFMVGRG
                  ***************:* ***************:**:*:* ***.**.********** *
```

```
Aae_Raf        KLRPDLTNVRSDCPQALKRIVEDCIKFSREDRPLFRLLLNMLENMLRTLPKFHRSASEPN
Aal_Raf        KLRPDLANVRSDCPQALKRIVEDCIKFGREDRPLFRLLLNMLENMLRTLPKFHRSASEPN
Cq_Raf         KLRPDLTKVRSDCPQALKRCVEDCIKYSREERPLFRLLLNMLENMLRTLPKFHRSASEPN
Ag_Raf         KLRPDSTKVRTDCPQALKRCVEDCIKFNRDERPLFRLLLNMLENMLRTMPKFHRSASEPN
Dm_Raf         LLRPDMSQVRSDAPQALKRLAEDCIKYTPKDRPLFRPLLNMLENMLRTLPKIHRSASEPN
                **** ::**:*.****** .*****:  .:***** ***********:**:********

Aae_Raf        FTQTQLQNDDFLYLCSSPKTPVNFHNFQFYNGTGNI
Aal_Raf        FTQTQLQNDDFLYLCSSPKTPVNFHNFQFYNGTGNI
Cq_Raf         FTQTQLQNDDFLYLCSSPKTPVAFHNFQFNNGFGNI
Ag_Raf         FTQTQLQNDDFLYMCSSPKTPVNFHNFQFYNGAGNY
Dm_Raf         LTQSQLQNDEFLYLPS-PKTPVNFNNFQFFGSAGNI
               :**:*****:***: * ***** *:**** .. **
```

Multiple sequence alignment of MEK

```
Aae_MEK        MSKMTKNKLNLTLPPGSVDVTASQQTT-PTPSFKTPSGTEYVIGKHNLLGKPKNSIDALT
Aal_MEK        MSKMTKNKLNLTLPPGSVDVTASQQTT-PTPSFKTPSGTEYVIGKHNLLGKPKNSIDALT
Cq_MEK         MSKMTKNKLNLTLPPGSVDVTASQQTT-PTPSFKTPSGTEYVIGKNNLLGKAKNSIDALT
Ag_MEK         MSKMTKNKLNLTLPPGSVDVPAGQQTT-PTPSFKTPSGTEYVIGKHNLLGKAKNSIDALT
Dm_MEK         ---MSKNKLNLVLPPVNTEATVAAATVAPTPPFKTPSGTD----THSLLGKPKTSIDALT
                  *:******.*** ..:....  *. ***.*******:    .:.****.*.******

Aae_MEK        ETLEELEIDENARKRIKVFLSQKEKIGELSDEDLEKLGELGSGNGGVVMKVRHIPTELIM
Aal_MEK        ETLEELEIDENARKRIKVFLSQKEKIGELSDEDLEKLGELGSGNGGVVMKVRHTPTELIM
Cq_MEK         ETLEELEIDENARKRIKVFLSQKEKIGELNDDDLDKLGELGSGNGGVVMKVRHIPTQLIM
Ag_MEK         ETLEELEMEESARKRIKVFLSQKEKIGELSDEDLEKLGELGSGNGGVVMKVRHIPTQLIM
Dm_MEK         ETLEGLDMGDTERKRIKMFLSQKEKIGELSDEDLEKLGELGSGNGGVVMKVRHTHTHLIM
               **** *:: :. *****:***********.*:**:******************  *.***

Aae_MEK        ARKLIHLEVKPAIKKQIIRELKVLHDCNFPHIVGFYGAFYSDGEISICMEYMDGGSLDLI
Aal_MEK        ARKLIHLEVKPAIKKQIIRELKVLHDCNFPHIVGFYGAFYSDGEISICMEYMDGGSLDLI
Cq_MEK         ARKLIHLEVKPAIKKQIIRELKVLHDCNFPHIVGFYGAFYSDGEISICMEYMDGGSLDLI
Ag_MEK         ARKLIHLEVKPAIKKQIIRELKVLHECNFPHIVGFYGAFYSDGEISICMEYMDGGSLDLI
Dm_MEK         ARKLIHLEVKPAIKKQILRELKVLHECNFPHIVGFYGAFYSDGEISICMEYMDGGSLDLI
               *****************:*******:**********************************

Aae_MEK        LKRAGRIPEPILAKITCAVLKGLSYLRDKHAIMHRDVKPSNILVNSSGEIKICDFGVSGQ
Aal_MEK        LKRAGRIPEPILAKITCAVLKGLSYLRDKHAIMHRDVKPSNILVNSSGEIKICDFGVSGQ
Cq_MEK         LKRAGRIPEQILAKITCAVLKGLSYLRDKHAIMHRDVKPSNILVNSSGEIKICDFGVSGQ
Ag_MEK         LKRAGRIPEAILAKITSAVLKGLSYLRDKHAIMHRDVKPSNILVNSSGEIKICDFGVSGQ
Dm_MEK         LKRAGRIPESILGRITLAVLKGLSYLRDNHAIIHRDVKPSNILVNSSGEIKICDFGVSGQ
               ********* **.:** ***********:***:***************************

Aae_MEK        LIDSMANSFVGTRSYMSPERLQGTHYSVQSDIWSLGLSLVEMAIGMYPIPPPDAKMLDYI
Aal_MEK        LIDSMANSFVGTRSYMSPERLQGTHYSVQSDIWSLGLSLVEMAIGMYPIPPPDAKMLDYI
Cq_MEK         LIDSMANSFVGTRSYMSPERLQGTHYSVQSDIWSLGLSLVEMAIGMYPIPPPDARTLDLI
Ag_MEK         LIDSMANSFVGTRSYMSPERLQGTHYSVQSDIWSLGLSLVEMAIGMYPIPPPDAKTLDLI
Dm_MEK         LIDSMANSFVGTRSYMSPERLQGTHYSVQSDIWSLGLSLVEMAIGMYPIPPPNTATLESI
               ****************************************************::  *: *
```

```
Aae_MEK         FQDKGDDSSPGQN-IIEPKPMAIFELLDYIVNEPPPKLEHHSFTDRFKDFVDRCLKKNPE
Aal_MEK         FQDKGDDSSPGQN-IIEPKPMAIFELLDYIVNEPPPKLEHHSFTDRFKDFVDRCLKKNPE
Cq_MEK          FQERPEDNSPGQSSLIEPKSMAIFELLDYIVNEPPPKLEHNSFSDRFKDFVDRCLQKNPE
Ag_MEK          FQERGEDCSPGQS-IIEPKPMAIFELLDYIVNEPPPKLEHNSFTDRFKNFVDLCLKKNPE
Dm_MEK          FADNAEESGQPTD---EPRAMAIFELLDYIVNEPPPKLEHKIFSTEFKDFVDICLKKQPD
                * :. :: .   .   **:.********************: *: .**:*** **:*:*:

Aae_MEK         ERADLKTLINHDWIKNIEQEDVDIAGWVCKTMDLLPSTPKRNASPN--
Aal_MEK         ERADLKTLINHDWIKNIEQEDVDIAGWVCKTMDLLPSTPKRNASPN--
Cq_MEK          ERADLKTLINHDWIKNIEQEDVDIAGWVCKTMDLLPSTPKRNASPN--
Ag_MEK          ERADLKTLMNHEWIKNIETEDVDIAGWVCKTMDLAPSTPKRNASPFSN
Dm_MEK          ERADLKTLLSHPWIRKAELEEVDISGWVCKTMDLPPSTPKRNTSPN--
                ********:.* **:: * *:***:********* *******:**
```

Multiple sequence alignment of ERK

```
Aae_ERK         MAEAAGQNNNAASAQAGPAG----ASNNGVTNTNPNAEVIRGQLFEVGPRYNQLAYIGEG
Aal_ERK         MAEAAGQNSNAACAQAGPAAGGGGTSNNGVTNTNPNAEVIRGQLFEVGPRYNQLAYIGEG
Cq_ERK          ------------------------------------------------------------
Ag_ERK          MAEAANNGAG--------------ASGSGANNNPNAEVVIRGQVFEVGPRYTNLAYIGEG
Dm_ERK          MEEFNSSGSVVN-------------GTGSTEVPQSNAEVIRGQIFEVGPRYIKLAYIGEG


Aae_ERK         AYGMVVSAVDTVTKTKVAIKKISPFEHQTYCQRTLREIKILTRFKHENIIDIRDILRVPS
Aal_ERK         AYGMVVSAVDTVTKTKVAIKKISPFEHQTYCQRTLREIKILTRFKHENIIDIRDILRVPS
Cq_ERK          --MLLFSTIYEWHANGVSL-----------------------------IIDIRDILRVPS
Ag_ERK          AYGMVVSAVDTVTKTKVAIKKISPFEHQTYCQRTLREIKILTRFKHENIIDIRDILRVPS
Dm_ERK          AYGMVVSADDTLTNQRVAIKKISPFEHQTYCQRTLREITILTRFKHENIIDIRDILRVDS
                   ::.*:        *::                             ********** *

Aae_ERK         IEQMKDVYIVQCLMETDLYKLLKTQRLSNDHICYFLYQILRGLKYIHSANVLHRDLKPSN
Aal_ERK         IEQMKDVYIVQCLMETDLYKLLKTQRLSNDHICYFLYQILRGLKYIHSANVLHRDLKPSN
Cq_ERK          IEQMKDVYIVQCLMETDLYKLLKTQRLSNDHICYFLYQILRGLKYIHSANVLHRDLKPSN
Ag_ERK          IEQMKDVYIVQCLMETDLYKLLKTQRLSNDHICYFLYQILRGLKYIHSANVLHRDLKPSN
Dm_ERK          IDQMRDVYIVQCLMETDLYKLLKTQRLSNDHICYFLYQILRGLKYIHSANVLHRDLKPSN
                *:**:*******************************************************

Aae_ERK         LLLNTTCDLKICDFGLARVADPEHDHTGFLTEYVATRWYRAPEIMLNSKGYTKSIDIWSV
Aal_ERK         LLLNTTCDLKICDFGLARVADPEHDHTGFLTEYVATRWYRAPEIMLNSKGYTKSIDIWSV
Cq_ERK          LLLNTTCDLKICDFGLARVADPEHDHTGFLTEYVATRWYRAPEIMLNSKGYTKSIDIWSV
Ag_ERK          LLLNTTCDLKICDFGLARVADPEHDHTGFLTEYVATRWYRAPEIMLNSKGYTKSIDIWSV
Dm_ERK          LLLNKTCDLKICDFGLARIADPEHDHTGFLTEYVATRWYRAPEIMLNSKGYTKSIDIWSV
                ****.*************:*****************************************

Aae_ERK         GCILAEMLSNRPIFPGKHYLDQLNHILGVLGSPSQEDLECIINEKARSYLQSLPYKPKVP
Aal_ERK         GCILAEMLSNRPIFPGKHYLDQLNHILGVLGSPSQEDLECIINEKARSYLQSLPYKPKVP
Cq_ERK          GCILAEMLSNRPIFPGKHYLDQLNHILGVLGSPSQEDLECIINEKARSYLQSLPYKPKVP
Ag_ERK          GCILAEMLSNRPIFPGKHYLDQLNHILGVLGSPSQEDLECIINEKARSYLQSLPYKPKVP
Dm_ERK          GCILAEMLSNRPIFPGKHYLDQLNHILGVLGSPSRDDLECIINEKARNYLESLPFKPNVP
```

```
                *********************************::***********.**:***:**:**

Aae_ERK         WSRLFPNADSNALDLLGKMLTFNPHNRISVEEALAHPYLEQYYDPADEPVAEEPFRIAME
Aal_ERK         WSRLFPNADSNALDLLGKMLTFNPHNRISVEEALAHPYLEQYYDPADEPVAEEPFRIAME
Cq_ERK          WSRLFANADPNALDLLGKMLTFNPHNRISVEEALAHPYLEQYYDPADEPVAEEPFRIAME
Ag_ERK          WSRLFPNADQNALDLLGKMLTFNPHNRISVEDALAHPYLEQYYDPADEPVAEEPFRIAME
Dm_ERK          WAKLFPNADALALDLLGKMLTFNPHKRIPVEEALAHPYLEQYYDPGDEPVAEVPFRINME
                *::**.***  **************:**.**:*************.****** **** **

Aae_ERK         LDDLPKETLKRLIFEETLRFNHNDNHPDVM-
Aal_ERK         LDDLPKETLKRLIFEETLRFNHNDNHPDAM-
Cq_ERK          LDDLPKETLKRLIFEETLRFNHNDNLPDSM-
Ag_ERK          LDDLPKETLKQLIFEETLRFNHND--PPV--
Dm_ERK          NDDISRDALKSLIFEETLKFKERQ--PDNAP
                 **:.:::** *******:*:..:  *
```
