# Supplementary material for: The Ras/ERK signaling pathway couples antimicrobial peptides to mediate resistance to dengue virus in Aedes mosquitoes
Source: PLoS Negl Trop Dis. 2020 Aug 31;14(8):e0008660. doi: 10.1371/journal.pntd.0008660 (PMC7485967; doi:10.1371/journal.pntd.0008660)
Supplement: S1 Table — (DOCX) [file pntd.0008660.s009.docx]

| **Target gene** | **Prime name** | **siRNA sequence** |
| --- | --- | --- |
| Aa.ERK | sense | 5'- GGAGACUGAUCUAUACAAATT -3' |
|  | antisense | 5'- UUUGUAUAGAUCAGUCUCCTT -3' |
| Aa.JNK | sense | 5'- GGGAAGGUGUGAAGUGGUUTT -3' |
| Aa.P38  Aa.Ras  Aa.CecB  Aa.CecC  Aa.DefC  Aa.Rel1  GFP control | antisense  sense  antisense  sense  antisense  sense  antisense  sense  antisense  sense  antisense  sense  antisense  sense  antisense | 5'- AACCACUUCACACCUUCCCTT -3'  5'- GCGGACCUGAACAAUAUUATT -3'  5'- UAAUAUUGUUCAGGUCCGCTT -3'  5'- CCGCAAAGACAAAGAAAGATT -3'  5'- UCUUUCUUUGUCUUUGCGGTT -3'  5'- GCAAAUCAGCAUCCAACCATT -3'  5-UGGUUGGAUGCUGAUUUGCTT-3  5'-CCAAGAUAUUCGUCCUGAUTT-3'  5'-AUCAGGACGAAUAUCUUGGTT-3'  5'-GCAUUCCCUGAAGAGCUGUTT-3'  5'-ACAGCUCUUCAGGGAAUGCTT-3'  5'-CCACACUACGGCAACACAATT-3'  5'-UUGUGUUGCCGUAGUGUGGTT-3'  5'- GGUGAUGCUACAUACGGAATT-3'  5'- UUCCGUAUGUAGCAUCACCTT-3' |
