# Supplementary material for: The Ras/ERK signaling pathway couples antimicrobial peptides to mediate resistance to dengue virus in Aedes mosquitoes
Source: PLoS Negl Trop Dis. 2020 Aug 31;14(8):e0008660. doi: 10.1371/journal.pntd.0008660 (PMC7485967; doi:10.1371/journal.pntd.0008660)
Supplement: S2 Table — (DOCX) [file pntd.0008660.s010.docx]

**S2 Table. The primer sequences for real time qPCR**

| **Gene ID** | **Primer name** | **Primer sequence** |
| --- | --- | --- |
| ***Aedes albopictus*(C6/36)** |  |  |
| LOC109417538 | qERK forward | 5'-CAAACACGAGAACATCATAGACATAAG-3' |
|  | qERK reverse | 5'- GTTCGCCGAGTGGATGTATTT-3' |
| LOC109429983 | qJNK forward | 5'- TACGCCTACTCACCGCAAAAC -3' |
|  | qJNK reverse | 5'- GCCGCACAGGATTTGATAGATT-3' |
| LOC109431243 | qP38 forward | 5'- GGGGCGGACCTGAACAAT-3' |
|  | qP38 reverse | 5'- GGACGGCTTCAAATCCCTGT-3' |
| LOC109406147 | qRas forward | 5'- AAATGTGATCTGCAGGCGTG -3' |
|  | qRas reverse | 5'- TGTAGAAGGCATCGTCAACTCC -3' |
| LOC109419598 | qSos forward | 5'- ACTGGATGGCGGATTTGGTTAT - 3' |
|  | qSos reverse | 5'- AACTCCCGTGCCTTCTCGC -3' |
| LOC109622896 | qDuox forward | 5'-GAATCTACTTCTGGTGGCTGTGC-3' |
|  | qDuox reverse | 5'-CAAGCCAAAAGCACCATCCC-3' |
| LOC109411712 | qREL1 forward | 5'-GGCAACCAAATGACTCTCCA -3' |
|  | qREL1 reverse | 5'-GGCTCCCAGCAGGTAATCC -3' |
| LOC109399595  LOC109416795  AF145802.1  AF145803.1  AF145804.1  LOC109399074  LOC109409889  AF087815.1  ***Aedes albopictus* (Foshan)**  AALF009980  AALF019289  AF145802.1  AF145803.1  AF145804.1  LOC109399074  LOC109409889  AF087815.1  ***Aedes aegypti* (Aag2)**  LOC5575808  LOC5568816  LOC5564226  **DENV2 (****New Guinea C)**  KM204118.1 | qRps7 forward  qRps7 reverse  qNos reverse  qNos reverse  qCec-A forward  qCec-A reverse  qCec-B forward  qCec-B reverse  qCec-C forward  qCec-C reverse  qDef-A forward  qDef-A reverse  qDef-C forward  qDef-C reverse  qDef-D forward  qDef-D reverse  qSos forward  qSos reverse  qRas forward  qRas reverse  qCec-A forward  qCec-A reverse  qCec-B forward  qCec-B reverse  qCec-C forward  qCec-C reverse  qDef-A forward  qDef-A reverse  qDef-C forward  qDef-C reverse  qDef-D forward  qDef-D reverse  qRas forward  qRas forward  qSos forward  qSos forward  qRps17 forward  qRps17 forward  qNS1 forward  qNS1 reverse | 5'-GAAGTTGTCGGAAAGCGTATGC -3'  5'-TTCAATGGTGGTCTGCTGGTTC -3'  5’- ACGCTCTACAGCCTCGTTTCTAC-3'  5’- GACTCCGTAGTGTTCGGCTCC -3'  5’-GCTTTGGTCCTGCTGGGTC -3'  5’-GAGCTTTCTCCGATGCTTTGA -3'  5’-TCTGCTTATCGGATTGGTGCT -3'  5’-ACTCGCTTGCCGACTCCTT -3'  5’-TCGGTCAGTCGGAAGCAGGT-3'  5’-TTCCAAGAGCCTTTGCACCAG-3''  5’- TGTGATTTGTTTCCTGGCTATG -3'  5’- CTGTCACCCACGCCGAAT -3'  5’- TCGGCAAACGCAAACCTT -3'  5’- GGATTCGGTGTTGGGGATAG -3'  5’-GTCTGTTGCCAACTCTCTTT-3'  5’-CACAAGCACTGTCACCAAC-3'  5'- GATGGCGGATTTGGTTATGC-3'  5'-GATCGGCGTAAATGTGGTAGG-3'  5'-TGGGAGTTGACGATGCCTTCT -3'  5'-GCCGACTGGAGCCAAGTTTATT -3'  5'-GCTTTGGTCCTGCTGGGTC -3'  5'-GAGCTTTCTCCGATGCTTTGA -3'  5'-TCTGCTTATCGGATTGGTGCT -3'  5'-ACTCGCTTGCCGACTCCTT -3'  5'-TCGGTCAGTCGGAAGCAGGT-3'  5'-TTCCAAGAGCCTTTGCACCAG-3'  5'- TGTGATTTGTTTCCTGGCTATG -3'  5'- CTGTCACCCACGCCGAAT -3'  5'- TCGGCAAACGCAAACCTT -3'  5'- GGATTCGGTGTTGGGGATAG -3'  5'- GTCTGTTGCCAACTCTCTTT -3'  5'- CACAAGCACTGTCACCAAC-3  5’ ' TAGATACAGCAGGACAAGAAGA -3'  5'- AACAAGCAAAAAGCCTTCACCAG -3’  5'- CGGAAGCGGCAGAGTTTGATGTGTA -3’  5'- GCCTGAAATAGATCGCATGGGTTAG -3’  5'-CACTCCCAGGTCCGTGGTAT -3'  5'- GGACACTTCCGGCACGTAGT-3'  5'- AAGATACAGAGGTGAGGACGGATG-3'  5'- GCTGTGACCAAGGAGTTGACCA-3' |
